# The Hartree–Fock Exchange for Crystalline Systems: The Implementation with an (*All-Electron*) Gaussian-Type Basis Set and Numerical Evidence with Reference to Perovskites

**DOI:** 10.1021/acs.jctc.6c00118

**Published:** 2026-04-29

**Authors:** Roberto Dovesi, Klaus Doll, Mauro Causà, Michel Rérat, Philippe D’Arco

**Affiliations:** † 66161 ACCADEMIA DELLE SCIENZE DI TORINO, Via Accademia delle Scienze 6, Torino (To) 10123 , Italy; ‡ 9149 University of Stuttgart, Molpro Quantum Chemistry Software, Institute of Theoretical Chemistry, Pfaffenwaldring 55, Stuttgart D-70569, Germany; § Piazza San Rocco 8, Giaveno (To) 10094, Italy; ∥ Université de Pau et des Pays de l’Adour, E2S UPPA, CNRS IPREM, 2 avenue du Président P. Angot, Pau F-64053, France; ⊥ Sorbonne Université, CNRS-INSU, Institut des Sciences de la Terre, ISTeP UMR 7193, Paris F-75005, France

## Abstract

The treatment of the Hartree–Fock exchange (HFX) series in periodic systems is described when a Gaussian-type basis set is adopted, and its performance is documented with reference to the KCrF_3_ perovskite. The computational scheme for the exchange, implemented in the CRYSTAL code in its general lines more than 35 years ago (Causà et al., J. Chem. Phys. 92, 909 (1988)), is here documented for the first time with reference to the variables essential for the description of many properties of periodic systems: truncation of the exchange series, reduction of point symmetry, change of space group, and dimension of the unit cell: these features are involved in the calculation of the total energy, the equilibrium geometry, the relative stability of phases, the Jahn–Teller splitting and orbital ordering, the ferromagnetic versus antiferromagnetic energy difference, the vibrational frequencies, and the IR and Raman intensities, to quote the most important properties of interest. The same computational scheme is used for both pure HF and for *full-range* or *range-separated hybrid* functionals, in which a variable percentage X of HFX is used. To document the role of X, a very recently defined functional is employed, PBE­(X), such that PBE(0) = PBE and PBE(25) = PBE0. The high efficiency of the algorithms implemented in CRYSTAL permits to perform calculations for supercells of KCrF_3_ containing, for example, 3430 atoms (7 × 7 × 7 supercell of the monoclinic cell, containing 10 atoms), when an *all-electron* basis set of triple-ζ quality is adopted.

## Graphical abstract

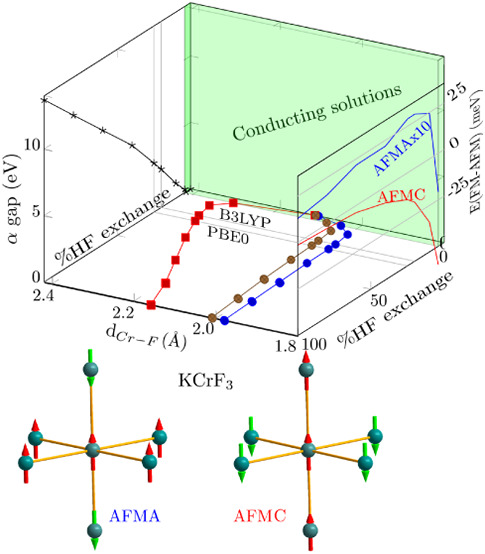

## 1 Introduction

The implementation of an accurate and efficient scheme for the evaluation of the Hartree–Fock exchange (HFX), as it appears in *full range* hybrids, like B3LYP or PBE0,^1–3^ *range separated* hybrids, such as HSE06,^4,5^ or in the HF scheme itself,^6,7^ is still a challenging task, as documented by the numerous recent papers referring to public, widespread computer codes (see for example Head-Gordon and collaborators^8^ in 2022, Subramanian et al.^9^ and Bussy and Hutter^10^ in 2024, and Pouillon et al.^11^ in 2026).

First attempts of formulations of quantum mechanical schemes for periodic systems in one, two or three dimensions date back to the early seventies, when many groups^12–15^ tried to generalize semiempirical or HF schemes for finite systems (molecules), at that time already very diffused. The most interesting and robust formulation which appeared in these years is due to Euwema et al.,^16–18^ who investigated with an HF scheme many properties (including the total energy, equilibrium geometry, band gap and Compton profiles) of 3D systems, such as diamond, LiF and Ne.

In 1980 Pisani and Dovesi^6^ formulated a HF scheme in the Gaussian basis for 3D,^19^ 2D^20^ and 1D^21^ systems. The theory behind was exposed in 1988 in a book,^7^ published in parallel to the first release of the associated code, CRYSTAL (overall, nine public releases have been distributed from 1988 to 2023).

In these years, simulation in solid state physics was dominated by computational schemes using a plane waves basis set, pseudopotentials for the inner electrons, and the simplest formulations of DFT, namely LDA and GGA. In the decade between 2000 and 2010, it became however evident that a fraction of the HFX is necessary for eliminating some of the drawbacks of pure DFT, *in primis* the very large underestimation of the band gaps of solids, that at that time was very often the most frequently investigated quantity. The formulation of the *range separated* variant of the *hybrid* functionals,^4,5^ already popular in the molecular context, raised the interest of many groups for the computation of the HFX series for periodic compounds.

It is in this context that, in 2008, the challenge was thrown, concerning the evaluation of the HF total energy *limit* of a simple crystalline system, LiH (cubic, two atoms in the primitive cell) by Gillan et al.^22^

The *LiH HF energy limit* challenge was accepted one year later by three groups, whose results were published in two papers.^23,24^ In Paier’s paper a large Gaussian-type basis set was used for approaching the HF LiH limit, by using two different schemes:

1 Thirty-one short-range screened-exchange HF calculations were performed with decreasing screening parameter ω (Gaussian suite of programs^25^). The results were then fitted and extrapolated to ω = 0, which corresponds to the unscreened *full range* HFX.
2 A truncated Coulomb operator, large supercells of increasing size, and the single **k** point in reciprocal space are the ingredients of the second approach (CP2K^26–28^ ); The *converged* supercell was as large as 1000 atoms (5 × 5 × 5 supercell times 8 atoms of the conventional cell).

So both schemes seem numerical exercices of very high accuracy, but characterized by an extremely high computational cost (31 different self-consistent field (SCF) calculations in the first case, a supercell with 1000 atoms in the second case). The VASP program^29,30^ and a plane waves basis set were used in the third approach. Paier et al. claim that similar high quality calculations *appear unlikely ... to ... be feasible using the current CRYSTAL code*.^23^ The statement originated from the observation that using more and more severe computational conditions (the TX parameters to be described in the next section) in the CRYSTAL06 code, the program was stopping, because one of the static dimensions of the code, defining a list of lattice vectors, sufficient in these years when small basis sets and loose computational conditions were used, was insufficient for the more severe conditions required by Gillan et al.^22^ One year later, the authors of the CRYSTAL code, stimulated by this challenge, removed the limit of this static dimension (it became dynamic, as most of the dimensions of vectors and matrices, from CRYSTAL09 on) and repeated the calculation for LiH, whose results are in Table 1 of a Comment^31^ to Paier’s paper,^23^ showing the absolute and monotonic convergence of the LiH total energy vs the TX tolerances. In short, the total energy obtained by the two schemes presented in Paier’s paper^23^ are −8.064543E_*h*_ (Gaussian) and −8.064545E_*h*_ (CP2K), to be compared with −8.064544E_*h*_ obtained with CRYSTAL^31^ (the difference is 1·10^–6^E_*h*_ with both!).

**Table 1:** The Basis Set Used for the Present Calculations^a^

	Shell number	Shell type	Exponent	Coeff s (or d)	Coeff p	Population
Cr	I	s	268186.0	0.000228		2.000
			38450.0	0.001929		
			8196.8	0.0111		
			2145.22	0.05		
			647.063	0.17013		
			223.658	0.3689		
			87.3526	0.4035		
			36.2695	0.1435		
	II	sp	673.598	–0.0055	0.0085	8.054
			160.774	–0.0685	0.0608	
			53.4355	–0.128	0.2122	
			21.1062	0.2526	0.3977	
			8.9184	0.6295	0.4003	
			3.1675	0.276	0.2146	
	III	sp	34.5046	0.023	–0.0234	2.155
			14.0107	–0.2664	–0.0747	
			5.5869	–0.8325	0.1897	
			2.5492	0.8768	1.2982	
	IV	sp	1.0884	1.0	1.0	4.327
	V	sp	0.4521	1.0	1.0	1.509
	VI	d	18.0911	0.0809		3.558
			4.9429	0.3265		
			1.6725	0.538		
			0.6396	0.4633		
	VII	d	0.2532	1.0		0.656
F	I	s	13770	0.000877		1.999
			1590.0	0.00915		
			326.5	0.0486		
			91.66	0.1691		
			30.46	0.3708		
			11.50	0.41649		
			4.76	0.1306		
	II	sp	19.000	–0.1094	0.1244	3.897
			4.530	–0.1289	0.5323	
			1.387	1.0	1.0	
	III	sp	0.440	1.0	1.0	3.018 (3.010)
	IV	sp	0.179	1.0	1.0	1.001 (0.997)
K	I	s	172500	0.000220		2.000
			24320	0.00192		
			5140	0.01109		
			1343.9	0.04992		
			404.5	0.1702		
			139.4	0.3679		
			54.39	0.4036		
			22.71	0.1459		
	II	sp	402.0	–0.00603	0.00841	8.078
			93.5	–0.0805	0.0602	
			30.75	–0.1094	0.2117	
			11.92	0.258	0.3726	
			5.167	0.684	0.4022	
			1.582	0.399	0.186	
	III	sp	17.35	–0.0074	–0.0321	3.957
			7.55	–0.129	–0.062	
			2.939	–0.6834	0.1691	
			1.19	1.08	1.500	
			0.674	1.03	1.060	
	IV	sp	0.389	1.0	1.0	2.628
	V	sp	0.216	1.0	1.0	1.346

a The sp GTFs share the same exponent, but the coefficients for the s and p functions are different. The shells are labelled with Roman numbers, in increasing order. There are 7, 4, and 5 shells for Cr, F and K, respectively. In the last column, the Mulliken population (in |*e*|) of the shell is indicated (HF, FM). For F, the populations refer to F_2_ (equatorial F). When the numbers for F_1_ (apical F) are different, they are given in parentheses. The atomic Mulliken charges are 22.259 (Cr), 9.915 (F_2_), 9.901 (F_1_) and 18.009 (K), corresponding to a net atomic charge of +1.741 (Cr), −0.915 (F_2_), −0.901 (F_1_), +0.990 (K).

The most noticeable difference among these approaches is in the computational costs: 200 to 800 s, according to TX, on a single core Intel Xeon E5440 2.33 GHz^31^ with CRYSTAL, to be compared with a cost which is not quantified in Paier’s paper, but appears to be orders of magnitude higher.

There is however a point which is of crucial importance in the above discussion: the schemes illustrated in ref 23 for obtaining the HF energy limit of LiH are NOT standard options of the applied codes, and can hardly be applied to more complicated unit cells, with, say, 20, or 200 atoms/cell, instead of 2, or to more demanding properties such as the vibrational spectrum. With CRYSTAL, e.g., calculations for MgO supercells with up to 10648 atoms were recently presented.^32^

As regards perovskites it is worth mentioning the studies published in recent years by some of the present authors on the two families, KBF_3_ and LaBO_3_, in which many properties have been investigated, by using B3LYP, HF and, in some cases, PBE0, HSE06 and PBE. In these studies very often unit cells of different size, shape and symmetry have been compared with high accuracy. Particularly relevant for the present discussion are two papers, one devoted to KFeF_3_,^33^ and the second one extending the analysis to the four t_2*g*_ Jahn–Teller distorted members of the family, namely KScF_3_, KTiF_3_, KFeF_3_, KCoF_3_.^34^ In these cases, the local symmetry on the transition metal (TM) is broken, and the occupied d orbital (for example d_*xy*_ in the Sc compound) is allowed to assume different orientations in different sites. So the problem was to tackle the many possible orientations in a large supercell, and their relative stability. In all cases a supercell of size 2 × 2 × 2 of the simplest tetragonal cell was considered, containing then 40 atoms and 8 TMs, and all possible different orientations have been enumerated. The total number of possible configurations is 6561, belonging to 162 classes of symmetry independent configurations, whose structure has been optimized at the B3LYP level, and the energies ordered.

Looking at the literature between 2010 and 2025, with reference to the treatment of the HF exchange for periodic systems, apparently the progress has been limited with respect to the situation described above and referring to the years 2008–2009.^22–24^ In the following we mention a few examples that seem to support this statement. We will concentrate on perovskites,^33,35,36^ as we chose this class of compounds as reference systems for illustrating our computational scheme.

In 2012 He and Franchini^37^ investigated with the VASP code^29,30^ and the PBE+U and HSE06 functionals, the electronic structure of nine LaBO_3_ compounds (B from Sc to Cu), using the experimentally determined structure.^38–50^ In a couple of cases, LaVO_3_ and LaCuO_3_, two different space groups have been proposed in the literature. The density of states, band structure, charge and spin distributions, equilibrium geometry of the two structures have been computed and compared; however the total energies (available from the geometry optimization) are not compared, and the relative stability not discussed. This seems to suggest that the adopted PBE+U and HSE06 schemes are not reliable enough for the comparison of the total energies, when a different number of atoms and point symmetry operators are involved.

In 2013 Scuseria et al.,^51^ for investigating LaTiO_3_, SrTiO_3_ and LaAlO_3_ with the GAUSSIAN code, adopted the HSE06 functional, because *the use of global hybrids ... remains limited due to their high computational cost*.

In 2019 Varignon et al.^52^ used pseudopotentials, a plane wave basis set, and the LDA+U and HSE06 functionals with the VASP code^29,30^ for investigating a series of perovskites, including LaTiO_3_, LaVO_3_ and LaMnO_3_. When using the LDA+U scheme, the optimization was performed with the unit cell containing 4 formula units (f.u.), i.e., twenty atoms. For HSE06, the optimization is limited to the primitive cubic cell with one f.u., for evident computational limits (see the caption of Table 1 of ref 52).

In the present manuscript we present the scheme implemented in the CRYSTAL code for HFX, and provide evidence of its effectiveness, using as test system the KCrF_3_ perovskite, whose main features are summarized in the next section.

First, we will document the accuracy of our HFX implementation with reference to five different properties computed at the HF level:

1 The total energy.
2 The equilibrium geometry.
3 The energy difference between three competing phases of KCrF_3_, corresponding to space group I4/mcm (N. 140), P4/mbm (N. 127), I112/m (N. 12).
4 The energy difference between the ferromagnetic (FM) and the antiferromagnetic (AFM) solutions.
5 The vibrational spectrum (wavenumbers and IR intensities).

Point I) is the same as discussed in the paper by Paier et al.;^23^ this point is however the least interesting, as it is well-known that the absolute total energy (i) has no experimental counterpart, and (ii) it is converging very slowly with respect to many computational parameters. Consider for example the convergence with respect to the basis set size in the core region, or in the inner part (with nodes) of the valence region, which can be extremely slow and expensive, but has no influence on most of the valence properties, if a reasonable, well calibrated, but certainly incomplete set is adopted; this is the reason why so frequently pseudopotentials are used. The quantities at points II), III), IV) and V) are on the contrary *differences*, so that many of the numerical inaccuracies (*in primis* the basis set incompleteness), cancel.

We will then extend the analysis to *hybrid* schemes, exploring the influence of X, the percentage of HFX in the functional. We use the PBE­(X) functional,^35,53^ such that PBE(0) = PBE and PBE(25) = PBE0. The results for X = 0, 20, 25, 40, 60, 80, and 100 have been obtained for the following properties:

1 The band gap.
2 The total energy difference between two tetragonal and the monoclinic solutions; this permits to quantify the various contributions (as a function of X) to the stabilization of the monoclinic structure: Jahn–Teller distortion, orbital ordering, CrF_6_ octahedra rotation.
3 The variation of the Cr–F distances in the *ab* basal plane.
4 The relative stability of the FM and AFM solutions (AFMA, AFMC and AFMG).

We anticipate that this analysis will show the important role of HFX in determining many of the interesting properties of these systems. This conclusion applies not only to the full set of perovskites, but also, more generally, to all ionic and covalent systems.

Along this analysis we will also document the high efficiency and accuracy of the proposed scheme: we will show that large unit cells containing transition metals can be treated at the *all electron* level with hybrid functionals. This will be documented by computing the HF total energy of various supercells of KCrF_3_ (with up to 3430 atoms) on relatively small machines (64 processors) in a reasonable CPU time.

The paper is structured as follows: The properties of KCrF_3_ are described in Section 2. In Section 3, the computational conditions are defined, and in Section 4, this discussion focuses on the implications of these conditions for the system KCrF_3_. The results section is divided in two parts, the first (Section 5) referring to pure HF, the second (Section 6) reporting data obtained with the PBE­(X) functional, with X spanning from 0 to 100, and with PBE0, HSE06, B3LYP, HF. The conclusions appear in Section 7.

## 2 The Structural, Electronic, and Magnetic Properties of KCrF_3_

KCrF_3_ is one of the few materials that possess a pseudocubic crystal structure and one-dimensional magnetic properties.^54–58^ This is a consequence of the d^4^ $\left(t_{2g}^{3}e_{g}^{1}\right)$ occupancy of Cr, and then of the interplay between the exchange interaction and the orbital-ordering effects associated with the cooperative Jahn–Teller distortion of the CrF_6_ octahedra. The low temperature SG is I112/m (N. 12), whereas between 80 and 250 K the proposed experimental SG is tetragonal (I4/mcm, N. 140). The electronic and magnetic situation is very close to the one of KCuF_3_,^59–61^ with d^9^ $\left(t_{2g}^{6}e_{g}^{3}\right)$ occupancy in Cu, for which, however, the proposed SGs at low and high temperature (up to about 1000 K) is a mixing of I4/mcm and P4/mbm (N. 127), see Figure 1.

**Figure 1:**
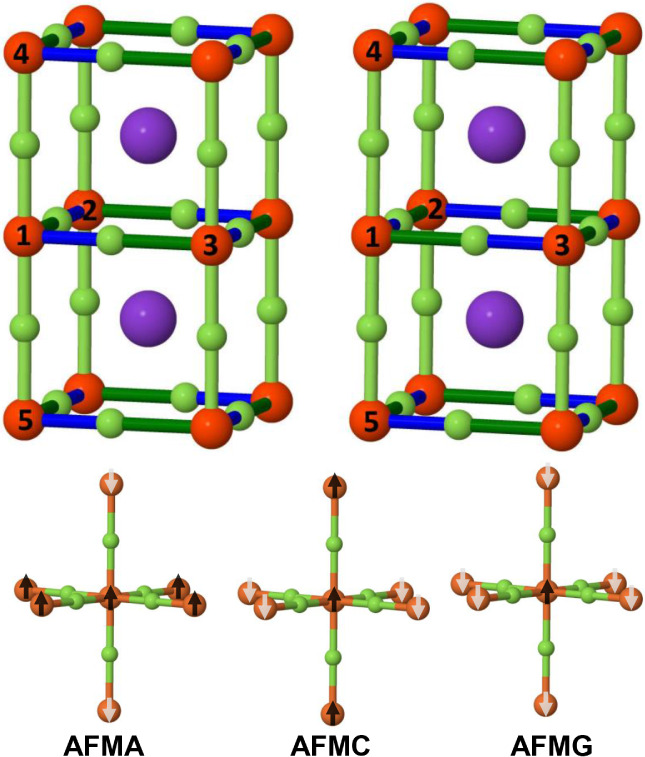
TOP: KCrF_3_ in the *untwisted* (P4/mbm, SG 127, left) and *twisted* (I4/mcm, SG 140, right) structure. The three Cr–F distances are different, as the different colors indicate. Along the *c* axis, F is midway between two Cr atoms (light green color). In the *ab* plane, the two Cr–F–Cr distances are different, and each Cr ion has two short (blue) and two long (dark green) bonds. In the *untwisted* structure, the situation is the same on the top, central and bottom plane. In the *twisted* case, short and long distances alternate when moving from plane to plane. BOTTOM: The three antiferromagnetic structures here investigated, with spin inversion along *c* (AFMA), in the *ab* plane (AFMC), and on all six first neighbors (AFMG).

The canonical e_*g*_ d orbitals ( $d_{2z^{2}-(x^{2}+y^{2})}$ and $d_{x^{2}-y^{2}}$ ) of Cr in KCrF_3_ are partially occupied; the obtained solution corresponds to an occupied $d_{z^{2}-x^{2}}$ or $d_{z^{2}-y^{2}}$ like orbital, according to the lattice position of the Cr ion in the *ab* plane. When moving along the x or *y* axis, the spin density alternates from one Cr atom to the next one due to the orbital ordering, and the periodicity is from one Cr to the second next; when moving along the *z* axis, the spin density remains the same for all Cr atoms and is periodic with a length from one Cr to the next one.

The Cr–F distances in the *ab* plane are not the same, due to the elongated shape of the Cr spin density, and can differ by as much as 0.34 Å (2.00 and 2.34 Å at the B3LYP level).

In the lowest part of Figure 1, the three AFM structures to be discussed in the following are sketched: AFMA, in which the spin on the two Cr ions along the *c* axis is reversed; AFMC, in which the four first neighbors in the *ab* plane are reversed; and AFMG, in which the spin of all the six first neighbors are reversed. An extensive analysis of the electronic and magnetic properties of KCrF_3_ has just appeared.^62^

## 3 Computational Aspects

The scheme illustrated here aims to provide a high numerical accuracy in the evaluation of:

1 The total energy and the gradient with respect to the inner coordinates and cell parameters;
2 The equilibrium geometry;
3 The energy differences between phases, characterized, in general, by a different size of the unit cell, different space group and different point symmetry, different magnetic order;
4 The vibrational frequencies, and the IR and Raman intensities.

It is clear that these steps are interrelated and that the accuracy of step 4 depends on step 2 and 1, and the accuracy of step 3 depends on step 1 and 2.

The Coulomb and Hartree–Fock exchange series in CRYSTAL are controlled by five parameters^63^ T1, T2, T3, T4 and T5. When not explicitly defined, T1, T2, T3 and T4 take the value TX and T5 = 2*TX, with TX = 10. The three exchange parameters (T3, T4 and T5) will be indicated in the following as Tx (lower case x). T1 and T2 refer to the truncation of the Coulomb series, and will not be discussed here: for a complete description of the role of T1 and T2, see refs 64, 65 and 66; the latter two, in particular, define the CRYSTAL strategy for the treatment of the Coulomb series for 3D systems^65^ and 1D systems.^66^ The algorithms for the 2D cases have been implemented in the same years (1980–1985), but have never been published.

The scheme is general and numerically very stable, so that the only difference in the actual use of the code with respect to these formulations is due to the increased speed of the computers, and to the implementation (at about the same years) of the *direct* strategy, according to which the integrals are computed at each cycle of the SCF, and not just at the beginning, and then stored on disk and read and used at each cycle. The two features allow us to use much more severe computational conditions: the calculations at the beginning of the eighties were run with TX = 4 (see below), whereas the default value for the present calculations is TX = 10.

T3, T4 and T5 are used for the truncation of the exchange series only, which will be discussed and documented in this manuscript. Also in this case the implementation, in its general lines, refers back to the eighties (see ref 67), but its formulation and accuracy has never been described and documented explicitly.

Also the scheme for the evaluation of the DFT exchange-correlation contribution to the Fock matrix has been implemented in the late nineties; the main features, and the parameters controlling the accuracy of the numerical integration over the unit cell volume are discussed in the CRYSTAL manual.^63^ In short, radial and angular points for the integration grid are generated through a Gauss–Legendre radial quadrature and Lebedev two-dimensional angular point distributions. In the present work, a pruned grid with 99 radial and up to 1454 angular points was used (see XXLGRID keyword in the CRYSTAL manual^63^).

### 3.1 The Basic Equations

Consider the contributions to the Fock matrix represented in direct space:

1 $$F_{\mu \nu }^{g}=h_{\mu \nu }^{g}+\underset{\lambda \rho }{\sum }\underset{l}{\sum }P_{\lambda \rho }^{l}\underset{h}{\sum }\left[\left(\mu^{0}\nu^{g}|\lambda^{h}\rho^{h+l}\right)-1/2\left(\mu^{0}\lambda^{h}|\nu^{g}\rho^{h+l}\right)\right]$$

where the first summand contains the monoelectronic, and the second the Coulomb and exchange bielectronic contributions; μ^**0**^ is the μ-th AO (atomic orbital) centered in the reference cell (by definition at (0,0,0) in terms of the three lattice parameters), ν^**g**^ is the ν-th AO centered in cell **g**, and so on. $P_{\lambda \rho }^{l}$ is the (translation invariant) density matrix connecting the λ and ρ AOs separated by the vector **l**. The vertical bar separates the functions attributed to electron 1 from the ones of electron 2, according to the standard notation. It represents the |**r**_1_ – **r**_2_|^–1^ Coulomb operator.

The density matrix is defined as

2 $$P_{\lambda \nu }^{g}=\int_{BZ}dk\underset{j}{\sum }\Theta \left(\epsilon_{F}-\epsilon_{j}\left(k\right)\right)a_{\lambda ,j}{\left(k\right)}^{*}a_{\nu ,j}\left(k\right)\times \exp\left(ik\times g\right)$$

with the Heavidside function Θ, the Fermi energy ϵ_F_ (or for insulators an energy in the interval between the occupied and unoccupied bands), and the integral running over the Brillouin zone (BZ). The integral gets replaced with a sum over **k** which extends to all the points in which the Fock matrix has been diagonalized, according to a commensurate net (with shrinking factor IS, and centered at Γ or **k** = **0**) adopted for sampling the reciprocal space. In the present case of KCrF_3_, a shrinking factor IS = 8 provides an energy stable to the sixth decimal figure for the various explored phases. The corresponding number of **k** points is 59 and 150 for the tetragonal and monoclinic phases (SG 140 and SG 12). In the unrestricted Hartree–Fock scheme (UHF), the density matrix P^α^ (P^β^) is obtained by limiting the sum over *j* to the set of occupied orbitals α (β). The total (spin) density matrix is the sum (difference) of the contributions α and β, built separately:

P^*tot*^≡P^α+β^ = P^α^ + P^β^.

P^*spin*^≡P^α–β^ = P^α^ – P^β^.

The total energy is obtained by saturating the three indices in the following expression:

3 $$E_{tot}=1/2\underset{\mu \nu }{\sum }\underset{g}{\sum }P_{\mu \nu }^{g}\left(h_{\mu \nu }^{g}+F_{\mu \nu }^{g}\right)$$

At each SCF cycle, the *direct space* Fock matrix is Fourier transformed to reciprocal space, and then diagonalized at each **k** point of the first Brillouin zone:

4 $$F_{\mu \nu }^{k}=\underset{g}{\sum }F_{\mu \nu }^{g}\times \exp\left(-ik\times g\right)$$

5 $$F^{k}A^{k}=S^{k}A^{k}E^{k}$$

**S** corresponds to the overlap matrix, **E** to the eigenvalues, and **A** the eigenvectors.

#### 3.1.1 The Tx Parameters for the Truncation of the Exchange Series

We can now describe the role of the T3, T4 and T5 exchange thresholds.

T3: when one (or both) of the two products μ^**0**^λ^**h**^ or ν^**g**^ρ^**h**+**l**^ is smaller than 10^–*T*3^, the integral is disregarded.

T4 and T5 try to mimic the behavior of the density matrix elements $P_{\mu \nu }^{g}$ (T4) and $P_{\lambda \rho }^{l}$ (T5) on the basis of the behavior of the corresponding overlap of the *adjoined Gaussians* (see below).

This is a relatively rough estimate, and can be checked by increasing the values of T4 and T5 in subsequent calculations, and looking at the variation of the total energy and of the other properties of interest. For ionic systems, as the present one (KCrF_3_), as well as for covalent compounds, the convergence is easily reached. For metallic systems it would require extremely high values of T4 and T5 due to the only algebraic decay of the density matrix. This behavior, however, may be fitted from the values at medium T4 and T5, and extrapolated to large distances. Another approach to have a faster decaying density matrix for metals would be applying finite temperature.^68^

A more careful strategy could be possible when a *direct* SCF strategy is used (the standard choice nowadays), as the extent of the density matrix can in this case be known on the fly during the SCF. This strategy is, however, not yet implemented in the code.

The reason for using T5 ≫ T4 is that T5 selects the exchange integrals during the SCF process. Disregarding bunches of integrals, also when they have a limited influence on the total energy, might destabilize the SCF process, with a progressive buildup of the related error. T4, on the contrary, is used only in the evaluation of the total energy.

#### 3.1.2 The Number of **K**-Points in Reciprocal Space and the SCF Convergence Threshold

Two other parameters are important for the quality of the SCF calculation:

1 The shrinking factor IS defining the number of **k** points in which the Fock operator is diagonalized. The IS parameter is not critical, as KCrF_3_ is insulating, and will not require further discussion.
2 The TOLDEE parameter controlling the convergence of the SCF cycle (the cycle stops when the energy difference between two subsequent cycles is smaller than 10^–*TOLDEE*^ E_*h*_). In the present case, TOLDEE = 8 has been used for the geometry optimization, and TOLDEE = 10 for the frequency calculations.

#### 3.1.3 The Adjoined Gaussian

In the selection of the integrals of the infinite Coulomb and exchange series, a shell of any type is represented by a single s type Gaussian (called the *Adjoined Gaussian*), whose exponent is equal to the exponent of the most diffuse Gaussian of the contraction of Gaussian Type Functions (GTF) defining the shell. The use of the adjoined Gaussian makes the selection extremely rapid, for two reasons:

1 The calculation of the overlap between two s type functions is very cheap;
2 the adjoined Gaussian represents many GFTs. For example, in a contraction of, say, 6 sp type GTFs, it represents the 6 × 4 = 24 different GTFs of the shell; similarly, for a contraction of 4 d type GTFs, it represents the 20 (4 × 5) different GTFs in the shell.

#### 3.1.4 The FIXINDEX Directive

From the above discussion concerning the five TX parameters, it is clear that when the geometry of the system is changing, it can happen that classes of integrals are disregarded (or new integrals are included), as is the case, for example, when the volume is increasing (reducing), or one bond distance is becoming larger (shorter). For this reason, since the early nineties, an option has been implemented in CRYSTAL,^63^ named FIXINDEX, that allows to freeze the number of integrals to be computed at various similar geometries, performing the selection of these integrals for geometry B by using the reference geometry A. Details and examples can be found in the CRYSTAL manual.^63^

### 3.2 The Role of the Density Matrix and Its Decrease with Distance for Ionic Compounds

Let us consider again the exchange contribution in eq 1, and look for the dominant terms at large distances between the origin cell and cell **g** at distance R­(**g**). The charge involved is maximum when **h** = **0** for electron one, and when **h** + **l** = **g** for electron two; being however **h** = **0**, we end up with **l** = **g**. This means that the two (*in principle*) infinite summations in the definition of F^**g**^ in eq 1, reduce to a single term. Besides that, λ = μ and ρ = ν give the maximum charge for electron 1 and electron 2. Obviously other terms are important, but we remind that the overlaps between the AOs decay exponentially with distance, and that different AOs centered on the same atom, characterized by different quantum numbers, are orthogonal. The dominant exchange contribution to the Fock matrix at large distance D becomes then

6 $$F_{\mu \nu }^{g}\left(D\right)=-1/2P_{\mu \nu }^{g}\left(\mu^{0}\mu^{0}|\nu^{g}\nu^{g}\right)$$

And the contribution to the total energy at large distance D becomes

7 $$E_{tot}\left(D\right)=-1/4\underset{\mu \nu }{\sum }\underset{g}{\sum }{\left(P_{\mu \nu }^{g}\right)}^{2}\left(\mu^{0}\mu^{0}|\nu^{g}\nu^{g}\right)$$

As the bielectronic integral appearing in eq 6 scales with the modulus of **g**, but the number of **g** vectors increases, in a 3D system, with the cube of this modulus, the sum would be divergent, if it was not extenuated by P^**g**^.

It has long been known that, for insulators, P^**g**^ decays exponentially with the distance, the coefficient in front of the distance being proportional to the band gap of the system (see ref 69 and ref 70 in ref 71). For metallic or semi metallic compounds, the decrease is much slower. For polymers, including polyacetylene, Monkhorst and Kertész^71^ and Surjan et al.^72^ formulated and used an empirical formula according to which the P^*g*^/P^*g*‑1^ ratio decreases as −W/(W+G), where W is the valence bandwidth, and G is the band gap.

It is important to explore in practice what happens for very ionic compounds such as the perovskites. Let us suppose that *large distance*, at which the density matrix is extremely small, means, say, 20, or 50, or 100 Å; we must then consider all the **g** vectors within a sphere with these radii. For the present system, with a unit cell (containing 10 atoms, 2 f.u.) with V = 154 Å^3^, the number of **g** vectors included in such a radius would be 152 or 812 or 6500.

But in eqs 1 and 3 the integrals involve three summations extended to all these cells, and then the number of exchange bielectronic integrals to be evaluated would be 152^3^, 812^3^ or 6500^3^ for each μ, ν, λ and ρ quadruplet. As each one of these indices spans the full set of AOs in the cell (83 per f.u.), in the worst case the number of exchange bielectronic integrals would be of the order of 10^20^. It is then clear that the tolerances Tx play a fundamental role.

The crucial point is then the extension of the density matrix. To this point we will devote the following tables and figures in the case of the KCrF_3_ perovskite. This analysis, which remains valid for semiconductors, should be reconsidered for the special cases in which the gap is null, like in metals, but also in graphite and symmetric polyacetylene. To the last two systems, an analysis similar to the present one^73^ will be devoted in the future.

### 3.3 Vibrational Frequencies, Infrared, and Raman Spectra

Frequencies at the Γ point are obtained within the harmonic approximation by diagonalizing the mass-weighted Hessian matrix, W, whose elements are defined as^74^

8 $$W_{\alpha i,\beta j}^{\Gamma }=\frac{H_{\alpha i,\beta j}^{0}}{\sqrt{M_{\alpha }M_{\beta }}}\;\mathrm{with}H_{\alpha i,\beta j}^{0}=\left(\frac{\partial^{2}E}{\partial u_{\alpha i}^{0}\partial u_{\beta j}^{0}}\right)$$

where *M*_α_ and *M*_β_ are the masses of the atoms associated with the coordinates *i* and *j* of atoms α and β. Energy first derivatives with respect to the atomic positions, *g*_*αj*_ = *∂E*/*∂u*_*αj*_, are calculated analytically for all the coordinates *u*_*αj*_ (*E* is the total energy, *u*_*αj*_ is the displacement coordinate with respect to equilibrium). Second derivatives at **u** = **0** are calculated numerically using a single displacement along each coordinate:

9 $$\left[\frac{\partial g_{\alpha j}}{\partial u_{\beta i}}\right]\approx \frac{g_{\alpha j}\left(0,...,u_{\beta i},...\right)}{u_{\beta i}}$$

For each displacement, an SCF+G (gradient) calculation is performed. The default value for the step *u* has been employed (0.003 Å). Infrared (IR) and Raman intensities are evaluated analytically through the CPHF scheme.^75–79^ The IR and Raman spectra can then easily be generated.

## 4 The Test Case: KCrF_3_

In this section we provide, first, numerical (indirect) evidence of the number of exchange bielectronic integrals evaluated, through the number of matrix elements of the density matrix *P* ^**g**^ involved in the calculation, and then, second, of the cost (in seconds) of the calculation for unit cells containing 5, 10, 20 atoms, when a single processor is used, or containing 80, 270, 640, 1250, 2160, and 3430 atoms, when a parallel calculation is performed using 64 processors. Preliminary to these two steps, is the presentation and discussion of the variational basis set adopted for the calculation.

### 4.1 The Basis Set and the Direct Lattice Vectors Involved in the Calculation

An *all electron* Gaussian type basis set, shown in Table 1, has been adopted, as for similar calculations on other KBF_3_ compounds.^36,80,81^ These basis functions will be indicated in the following as AOs; they are the result of a contraction (a linear combination of functions with fixed coefficients) of GTFs. The contractions of Table 1 are 8–6411(41) for Cr (the first function is a contraction of 8 GTF of s type, followed by 4 contractions of 6, 4, 1, 1 GTFs of s and p type, sharing the same exponent; 41 in parentheses indicates 2 contractions of d type, with 4 and 1 GTF); for F and K, the contractions are 7–311 and 8–6511. The total number of variational functions (AOs) in the basis is then 27 (Cr), 13 (F) and 17 (K), and the number of AOs per f.u. is 83; in the three cells here discussed, with two f.u./cell, the AOs are then 166 for the FM solution. Supercells with 4 f.u. have been used to generate the AFM solutions (AFMA, AFMC, AFMG), with 332 AOs/cell.

It is useful to analyze the basis set with some details, as it is crucial for the following discussion concerning the cost and accuracy of the evaluation of the exchange series (and more generally of the total energy).

The basis set is of *split valence* quality, indicating that the valence electrons are described by 3 sp shells, whereas the core electrons, which are supposed to remain essentially unaltered in various molecular or crystalline contexts, are described by long contractions, reproducing the shape of the core orbitals in the isolated atoms. The last column of the table gives the HF Mulliken population of the various shells (labeled with Roman numbers) for the SG 140 structure. So shell I of the three atoms (describing the 1s electrons) contains 2.000, 1.999, and 2.000 |*e*|; shell II, describing the 2sp electrons, contains 8.054 (Cr) and 8.078 (K) electrons (the ideal occupancy being 8 |*e*|). To the valence shells (for the 2sp electrons in F and the 3sp electrons in Cr and K) a much larger variational freedom is allowed, with three sets of sp type functions. The FM HF solution attributes 7.991 |*e*| to Cr, 7.931 |*e*| to K and 7.916 |*e*| to F_2_ (the four first neighbors of Cr in the *ab* plane) and 7.902 |*e*| to F_1_ (the two first neighbors of Cr along *c*), confirming the very ionic nature of the compound. As regards the Cr d shell, it contains 4.214 |*e*|, so that the total charge of Cr is 22.259 |*e*| (net charge of +1.741 |*e*|). For K the total number of electrons is 18.009, and the net charge is +0.991 |*e*|. For F, the total number of electrons is 9.915 (F_2_) and 9.901 (F_1_) with a net charge of −0.915 and −0.901 |*e*|, respectively. Obviously the sum of the four charges Q­(Cr) + Q­(K) + Q­(F_1_) + 2Q­(F_2_) ensures the electroneutrality of the cell, a crucial condition for the long-range electrostatics.

As aforementioned, in the selection of the integrals of the infinite Coulomb and exchange series, a shell of any type is represented by its *adjoined Gaussian*, whose exponent is equal to the exponent of the most diffuse Gaussian of the contraction of GTFs defining the shell. Table 1 shows that

1 Long contractions (up to 8 GTF) have an adjoined Gaussian with high exponent, so that in the selection of the integrals only a few cells of the infinite lattice are involved (actually in many cases only 1 cell is involved). This is evident from Table 2, where the *extension* of the shell–shell interaction for a set of diagonal matrix blocks is shown. In particular, all the diagonal blocks for Cr and F are listed, whereas for K only the blocks involving the most diffuse functions are shown. For the first four shells of Cr, only one lattice vector of the infinite set of **g** vectors (**g** = **0**) is involved. Looking at the column with Tx = 10 (the default value for the present calculations), we see that this number increases to 3 (only 13 **g** vectors, see the number in parentheses) for the diagonal elements involving shells V and VI; only for the most diffuse d function, this number increases to 5 stars of **g** vectors. For the most diffuse diagonal couples of F this number increases to 7.
2 The numbers in Table 2 clearly show why the cost of an *all electron* calculation with CRYSTAL is only marginally higher than a pseudopotential calculation (core electrons are very cheap).
3 The most diffuse shells (the expensive part of the calculation) are always represented by a single GTF, resulting in a low computational cost.

**Table 2:** Effect of the Exchange Tolerances on the Number of Stars of Direct Space g Vectors Included in the Calculation for KCrF_3_ (Hartree–Fock FM Solution, SG I4/mcm)^a^

Tx		6	10	16	22	AG
Cr	I–I (s)	1 (1) 0	1	1	1	36.27
	II–II (sp)	1	1	1	1	3.167
	III–III (sp)	1	1	1	1	2.549
	IV–IV (sp)	1	1	3 (13) 11.60	3	1.088
	V–V (sp)	1	3	5 (19) 16.40	7 (43) 19.94	0.452
	VI–VI (d)	1	3	3	5	0.640
	VII–VII (d)	3	5	9 (55) 23.19	13 (87) 27.99	0.253
F	I–I (s)	1	1	1	1	47.6
	II–II (sp)	1	1	1	3	1.38
	III–III (sp)	2 (9)	3	5	7	0.440
	IV–IV (sp)	5	7	13	19 (149) 33.42	0.179
K	V–V (sp)	4 (15) 15.67	7	12 (79) 25.93	16 (135) 30.59	0.216
						
F^**g**^		63476	63476	130816	206644	
F^**g**,*irr*^		8217	8217	14773	21592	
Ratio		7.72	7.72	8.85	9.57	
Range F^**g**^		12.37	15.67	20.14	23.79	
P^**g**^		160410	354134	719322	1156358	
P^**g**,*irr*^		10005	19083	35116	53362	
Ratio		16.03	18.56	20.48	21.67	
Range P^**g**^		17.46	22.65	28.67	33.59	
N of **g**		19	43	87	119	

a A star is the set of direct space vectors characterized by the same modulus. In parentheses, the number of **g** vectors belonging to the indicated stars of vectors is given; the largest modulus (in Bohr) is also shown. Examples refer to diagonal shell–shell blocks. For Cr and F all the possible diagonal blocks are shown; for K, for space reasons, only the one corresponding to the most diffuse shell. In the bottom part of the table the overall dimension of the matrices F**^g^** and P**^g^** is reported. *irr* indicates the symmetry *irreducible* part of the matrix, the one which is explicitly computed. Ratio stands for F**^g^**/F^**g**,*irr*^ and P**^g^**/P^**g**,*irr*^. The last line indicates the number of direct lattice **g** vectors involved in the calculation of the exchange series (we can call it the *exchange cluster*). AG is the adjoined Gaussian (in atomic units, Bohr^-2^).

In the bottom part of the table, the overall dimension of the *P* ^**g**^ and *F* ^**g**^ matrices are shown. In the table also the dimension of the irreducible part of these P and F matrices is shown. P^*irr*^ and F^*irr*^ contain the elements that are explicitly computed. P and F are generated from P^*irr*^ and F^*irr*^ by applying the point symmetry operators (which are 16 in the case of SG 140, adopted for extracting the numbers from Table 2). The ratio F/F^*irr*^ tends to the number of point symmetry operators, the ratio P/P^*irr*^ is above this number because for this matrix also the hermiticity $P_{\mu ,\nu }^{g}=P_{\nu ,\mu }^{-g}$ is exploited (for more details on the exploitation of symmetry, see refs 82,83 ).

Finally, the last line of the table quotes the maximum number of direct lattice vectors involved in the selection of the exchange integrals (the *exchange cluster*). It is clear that all these numbers increase dramatically with Tx (and obviously the number of bielectronic integrals increases to a higher power). However, as we will show in the following sections, at the Tx = 10 level of accuracy, all the important properties (*in primis* the total energy) are already very stable.

### 4.2 The Density Matrix Elements as a Function of the Distance from the Origin Cell

In this section we provide examples of the numbers appearing in the matrix P^**g**^, for core–shells, in Table 3, and for valence shells, in Table 4.

**Table 3:**
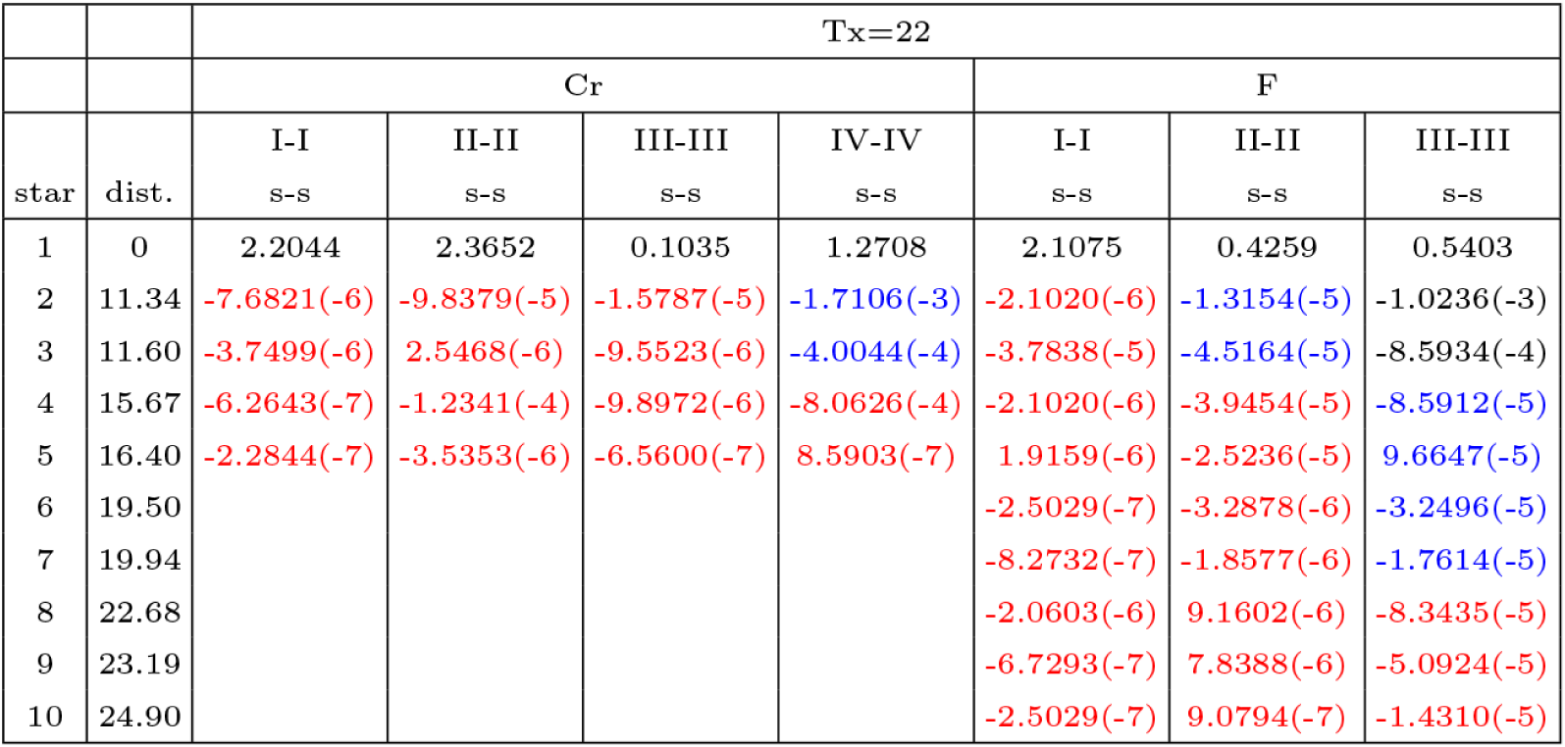
Diagonal Core Elements of the Direct Space Density Matrix P**^g^** for **g** Vectors Belonging to Stars (First Column; a Star is the Set of Direct Space Vectors Characterized by the Same Modulus) of Increasing Modulus (in the Second Column)^a^

a x.y­(−z) stands for x.y·10^–z^. For the selected shell couples, the number of computed matrix elements is different, as Table 2 shows. When the label of the star increases, the absolute value of the density matrix decreases. The SCF calculation is performed with Tx = 22, as indicated in the top line; this generates the black and blue numbers in the various columns. Repeating the classification (NOT the SCF calculation) with Tx = 10, the black numbers are selected (they coincide with the blue ones). Starting from the eigenvectors obtained with Tx = 22, it is possible, by Fourier transform of the reciprocal space eigenvectors (see eq 2) to obtain *a posteriori* additional elements of the density matrix, some of which are shown in red. Distance in Bohr.

**Table 4:**
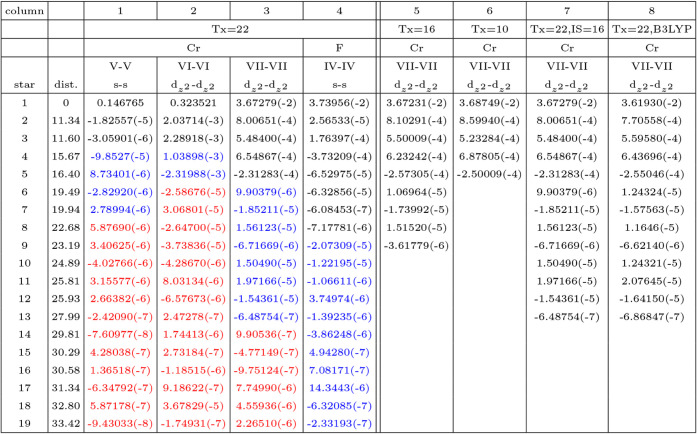
Diagonal Elements of the Direct Space Density Matrix P**^g^** for **g** Vectors Belonging to Stars of Increasing Modulus (in the Second Column, in Bohr, HF Calculations)^a^

a A star is the set of direct space vectors characterized by the same modulus. x.y­(−z) stands for x.y·10^–z^. Only valence elements are shown, as defined in Table 2. The SCF calculation is performed with Tx = 22, as indicated in the top line; this generates the black and blue numbers in the various columns. Repeating the classification (NOT the SCF calculation) with Tx = 10, the black numbers are selected (they coincide with the blue ones). Starting from the eigenvectors obtained with Tx = 22, it is possible, by Fourier transform of the reciprocal space eigenvectors (see eq 2) to obtain *a posteriori* additional elements of the density matrix, some of which are shown in red. The effect on the density matrix elements of performing the SCF cycle with Tx = 10 and Tx = 16 is shown in columns 5 and 6, to be compared with column 3. The effect of the functional (B3LYP vs HF) is shown by column 8 vs column 3, whereas column 7 (IS=16) vs column 3 (IS=8, value used in all other columns) documents the stability with respect to the shrinking factor.

Table 3 has been obtained running the SCF cycle with Tx = 22. With the converged solution, a much larger set of density matrix elements has been computed (red numbers) to show the evolution of the elements of P^**g**^ when the distance between the two involved centers increases. The black numbers correspond to Tx = 10, the blue ones to Tx = 22, and the red ones to Tx > 22. The value of the first excluded 1s–1s and 2s–2s element of the P matrix of Cr and F are as small as 10^–5^; similar comments apply to the other diagonal couples.

One should discuss how these small (disregarded, the red ones) numbers (a very large number of them), multiplied by the corresponding bielectronic integrals, contribute to the matrix F^**g**^, and how the latter, once diagonalized, contributes to the final total energy and related properties. We will obviously skip these intermediate (and cumbersome) steps. But Tables 7–9, and all the figures in this article, document how stable the final results are when tolerances from Tx = 10 are used.

In summary, Table 3 confirms that the *all electron* calculation with the CRYSTAL code is very cheap, because the 1s and 2sp electrons of Cr and K, and the 1s electrons of F require the calculation of a very small number of bielectronic integrals.

The situation is obviously different for the valence part, whose density matrix elements are shown in Table 4. The meaning of the three colors is the same as in the previous Table: red numbers, beyond Tx = 22; blue, between Tx = 10 and Tx = 22; black, up to Tx = 10. Note, however, that all these numbers are extracted from an SCF calculation with Tx = 22.

The most diffuse adjoined Gaussian is the one of shell IV of F, 0.179 Bohr^–2^, which requires the inclusion, for Tx = 22, of 19 stars of **g** vectors, up to a distance of 33.42 Bohr. Table 2 shows that 149 **g** vectors belong to these 19 stars. This means that in eq 1 the indices **h**, **l**, and **g** span (each) the interval 0–149, producing a huge amount of exchange integrals for the IV–IV shell couple. For the default value, Tx = 10, the number of stars reduces to 7 (the **g** vectors involved are *only* 43, see Table 2), as the blue color in Table 4 shows.

The last four columns in Table 4 provide additional information on the stability of the numbers in the table:

1 When the full SCF is performed with Tx = 10 or Tx = 16 (columns 6 and 5, respectively), does the density matrix (and then all the related properties) change? We reported only the VII–VII elements, to be compared with column 3; the element corresponding to star 5 changes from −2.50 × 10^–4^ (Tx = 10) to −2.57 × 10^–4^ (Tx = 16) to finally −2.31 × 10^–4^ (Tx = 22). These changes have, however, a limited effect on the final properties, as Tables 7–9 document.
2 The construction of the density matrix in direct space is the result of the Fourier transform of the product of the eigenvectors in reciprocal space (see eq 2); the Fock matrix in turn is antitransformed to reciprocal space (see eq 4) before diagonalization. At each cycle we go from direct to reciprocal space, and *vice versa*. This means that the accuracy of the P^**g**^ matrix might depend on the sampling in reciprocal space. Column 7 has been obtained using a shrinking factor IS = 16 for the reciprocal space sampling. The numbers are however extremely similar to the ones obtained with IS = 8 (column 3).
3 Finally, in column 8 the density matrix obtained with B3LYP is shown. The comparison with column 3 shows that the differences between the density matrices at the HF and B3LYP level are quite small, in spite of the large difference observed in a quantity that is usually adopted to judge the chemical character of the system, namely the band gap, which is about 14 eV for HF and 3 eV for B3LYP.

In summary, the two Tables 3 and 4 confirm that the density matrix elements of an ionic system like KCrF_3_ decrease rapidly (extremely rapidly in the case of core–shells) with the distance between the involved centers. The selection criterion based on the overlap of the adjoined Gaussian, although quite rough, ensures a reasonable efficiency in the truncation of the exchange series.

### 4.3 The Cost of the Exchange Bielectronic Integrals

In this section we evaluate the cost of the exchange series for periodic systems, and show that HF and hybrid calculations are possible and cheap for unit cells containing 10–20 atoms (the maximum size of interest for simple perovskites like KCrF_3_, here used as test system) on a single core processor, and for supercells containing up to 3430 atoms on larger computers, with the total energy per f.u. constant to the sixth decimal figure. Let us summarize the main steps of the SCF calculation in CRYSTAL, which are:

1 Preliminary calculations (outside the SCF cycle).
2 The construction of the density matrix P at cycle N, according to eq 2, by Fourier transforming the eigenvectors of cycle N-1. This step is indicated as PG in Tables 5 and 6.
3 With the matrix P^**g**^ (in vector form), the charges and multipoles of the shells are evaluated, by combination with the overlap matrix elements, the dipole matrix elements, and so on, up to the fourth order (L = 4) terms (this is the default value in CRYSTAL, although up to L = 6 can be used). Multipoles are in spherical harmonic form. POLES step.
4 Calculation of the bielectronic exchange and Coulomb integrals (the latter in the so-called *bielectronic zone*, to be complemented with the multipolar zone summed to infinity through the Ewald technique, described in the next step). BIELET step.
5 Calculation of the Coulomb series, in the multipolar expansion form, up to infinity (EWALD step).
6 Fourier transform of the Fock matrix to reciprocal space for each irreducible **k** point of the first Brillouin zone; then use of the symmetry to block factorize the matrix F^**k**^, and then diagonalization of eack symmetry block at each **k** point (FK step). Then the control goes back to point 2) for the next SCF cycle.
7 Final calculations outside the SCF cycle, including the calculation of some of the physical properties.

**Table 5:** Cost of the Calculation, in Seconds, of the B3LYP FM Ground State of KCrF_3_ for Cells of Different Size and Symmetry^a^

Column	1	2	3	4	5	6	7
N. atoms	5	10				10	20
SG	Pm3̅m	I4/mcm				I112/m	I4/mcm
NK	35	59				150	59
Tx	10	6	10	16	22	10	10
BIELET	11.5	12.1	20.9	43.8	68.6	70.1	70.3
DFT	0.1	1.0	2.5	2.9	3.1	7.3	7.3
EWALD	0.6	0.2	0.2	0.3	0.3	0.6	0.6
FK	0.1	0.7	0.8	0.8	0.8	4.3	4.3
PG	0.2	0.7	0.8	1.1	1.4	2.2	2.2
Total SCF	214	276	447	838	1253	1456	1452

a In column 1, the calculation refers to SG Pm-3m (cubic, 5 atoms/cell, N. 221). In columns 2, 3, 4, 5 the effect of Tx on SG I4/mcm (10 atoms cell, N. 140) is explored. Column 6 refers to the monoclinic I112/m group, so that the comparison of columns 3 and 6 shows what happens when the number of point symmetry operators reduces from 16 to 4. Finally, in column 7 the data of a double cell of SG I4/mcm are reported, so that by comparing columns 3 and 7 we can appreciate the increase of cost when the cell doubles. The number of atoms, the adopted SG, the number NK of **k** points are also indicated. The cost for the five dominant steps of the calculation (as defined in the CRYSTAL code, see text) within an SCF cycle is reported. Total SCF is the total cost required to reach convergence in the SCF calculation (about 16 cycles; we use 16 in all cases to make the comparison easier). The difference between this number and the sum of the five partial times multiplied by 16, is due to the initial and final calculations, outside the SCF cycle. The calculations are performed with a single processor desktop (Intel­(R) Core­(TM) I5-8265U, produced in 2018).

**Table 6:** Cost (in Seconds) of the Calculation of the HF FM Ground State of KCrF_3_ for Supercells (of the I112/m Cell with 10 Atoms) of Increasing Size, Up to 7 × 7 × 7, Corresponding to 3430 Atoms^a^

Column	1	2	3	4	5	6	7	8 (compare with 2)	9 (compare with 3)
Supercell	1 × 1 × 1	2 × 2 × 2	3 × 3 × 3	4 × 4 × 4	5 × 5 × 5	6 × 6 × 6	7 × 7 × 7	2 × 2 × 2	3 × 3 × 3
N. atoms	10	80	270	640	1250	2160	3430	80	270
N. AOs	166	1328	4482	10624	20750	35856	56938	1328	4482
SG	I112/m	I112/m	I112/m	I112/m	I112/m	I112/m	I112/m	I112/m	I4/mcm
N. SYM	4	4	4	4	4	4	4	4	8
IS	8	4	2	2	1	1	1	8	2
Nk	150	24	6	6	1	1	1	150	4
BIELET	2.8	21.0	87.4	213.2	438.2	834.2	1491.1	21.0	34.65
EWALD	2.0	5.6	6.9	28.1	91.8	248.2	733.0	5.5	4.8
FK	0.0	20.1	12.7	119.3	133.1	577.3	2151.2	73.1	18.7
PG	0.1	7.6	5.6	78.5	88.1	420.6	1714.9	22.3	5.5
ETOT/f.u.	–3882.272849	–3882.272850	–3882.272849	–3882.272850	–3882.272849	–3882.272850	–3882.272949	–3882.272849	–3882.272608
tSCF (cycles)	131 (24)	1676 (28)	2775 (22)	13070 (23)	29963 (28)	109778 (31)	343756.5 (30)	3654 (27)	2145 (32)
COULOMB	0.3	3.0	13.8	37.2	90.6	182.9	340.2		
EXCHANGE	1.7	18.0	73.6	176.0	347.6	651.3	1150.9		
DFT	0.4	1.0	3.8	11.7	67.3	138.3	277.5		

a Calculations performed at the geometry optimized for the 1 × 1 × 1 cell. Sixty-four cores of the Pyrene cluster of UPPA (large memory bigmem02-05 nodes). The cost for the five dominant steps of the calculation (as defined in the CRYSTAL code, see text) within an SCF cycle is reported. tSCF is the time required to reach an SCF convergence of 10^–7^ E*_h_* (energy difference between cycles N and N-1). The number of cycles is shown in parentheses. The difference between tSCF and the sum of the five partial times multiplied by the number of cycles, is due to the initial and final calculations, outside the scf cycle. The comparison of columns 8 and 2 shows the differences when IS = 8 or 2 is used for the 2 × 2 × 2 supercell. Columns 9 and 3 show the effect of the different number of symmetry operators (16 vs 4) for the 3 × 3 × 3 supercell. The total energy difference per formula unit between SG 12 and SG 140 for the 3 × 3 × 3 supercells is exactly the same obtained with the simple cells containing 10 atoms. The numbers in the last three rows are obtained from PBE calculations, providing the DFT data. Moreover, in this case the BIELET time includes only the COULOMB contribution, so that by difference with the BIELET line above the EXCHANGE contribution can be obtained.

We will limit the analysis to the steps in the SCF cycle, which is repeated many times (say 15 to 30 cycles are in general sufficient to satisfy the standard SCF convergence tolerance, 10^–8^ E_*h*_ total energy difference between cycles N and N-1).

Table 5 provides information and cost (in seconds) for a calculation in which a single core is used. The cost of step 3, POLES, is always negligible, and is not reported. So the dominant steps are 5: BIELET, EWALD, DFT (for a non pure HF calculation), FK, and PG, and are shown in the 5 central lines of the table. The most relevant comments are the following:

1 The calculation of the bielectronic integrals is the most expensive part of the calculation. It includes both the Coulomb and the exchange integrals. Performing a pure DFT calculation, the BIELET seconds are only due to the Coulomb integrals. When the conditions change from those of column 3 to those of column 2, the BIELET cost reduces from 20.9 to 12.1 s; then the exchange part of the calculation is in this case about 90% of the total cost.
2 At fixed SG (I4/mcm), the cost of the bielectronic part (Coulomb plus exchange) increases from 12 to 69 s when Tx increases from 6 to 22; for the tolerance used as default in the present work, Tx = 10, it is 21 s, while the cost of the other four steps is smaller than 5 s.
3 The DFT entry gives the cost (in seconds) of the DFT exchange-correlation functional in B3LYP. For Tx = 10, it is about 10% of the cost of a single SCF cycle.
4 The point symmetry plays obviously an important role: the total SCF cost for the tetragonal (16 symmetry operators) and monoclinic (four symmetry operators) cases is 447 and 1456 s respectively (10 atoms/cell, compare columns 3 and 6).
5 The cost of doubling the cell (from 10 to 20 atoms, columns 3 and 7) is about the same as the one for reducing the point symmetry from 16 to 4 point symmetry operators. The cubic case (column 1) with only 5 atoms/cell and 48 point symmetry operators, is obviously the cheapest calculation.

Overall, the cost of a complete SCF is between 400 to 1400 s; a full optimization, with say 20 steps, costs from 8000 (<2.5 h) to 28000 (8 h) of wall time on a single processor machine.

We discuss now the results obtained on a supercomputer, using 64 processors of the BIGMEMO3 machine of the MCIA Aquitaine mesocenter. The cost, in seconds/cycle for the various steps of the SCF calculation, and for supercells of increasing size, up to 7 × 7 × 7 (3430 atoms, 56938 AOs) are shown for the HF FM monoclinic case (SG 12) in Table 6 and in Figure 2. We list here the most relevant observations:

**Figure 2:**
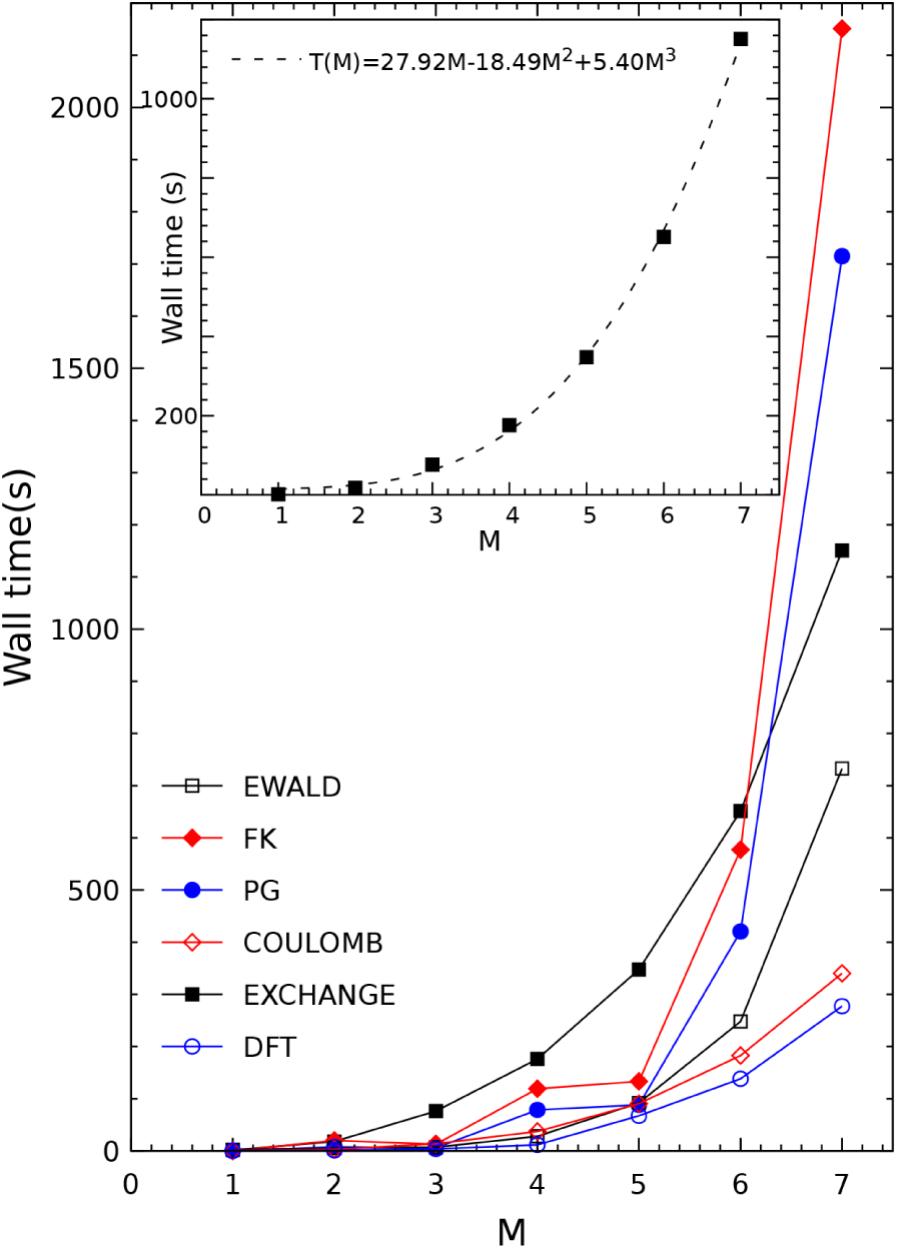
The cost (in seconds) for the six main steps (as described in the text, and listed in Table 6) of the SCF calculation for supercells (M, M, M) of KCrF_3_ (SG 12, FM) of increasing size (M = 1 to 7). The largest cell corresponds to 3430 atoms/cell. In the inset, the EXCHANGE data have been fitted with a third power of the supercell label M. Calculations performed with the parallel version of the code, by using 64 processors.

1 When larger and larger supercells are used, the cost of the diagonalization grows rapidly (Table 6, line FK), and becomes dominant from 6 × 6 × 6 on. We remind that the diagonalization of a matrix scales with power between 3 and 4 of the size of the matrix, whereas the cost of the bielectronic integrals scales with power three (see below). This trend of the diagonalization is compensated from 1 × 1 × 1 to 5 × 5 × 5 by the reduction of the number of **k** points in which the Fock matrix is diagonalized. Doubling the size of the cell as in going from 1 × 1 × 1 to 2 × 2 × 2, and from 2 × 2 × 2 to 4 × 4 × 4, permits to reduce the shrinking factor IS by a factor 2 at each step. In columns 2 and 8 the 2 × 2×2 supercell results are compared, when IS = 8 (150 **k** points) and IS = 4 (24 **k** points), are used. The diagonalization time per cycle reduces from 73 to 20 s. The total energy per f.u. is however the same to the sixth decimal figure (see table), showing that IS = 4 is sufficient for a very accurate sampling of the reciprocal space. Along the same line, for 3 × 3 × 3 and 4 × 4 × 4 we used IS = 2, and for 5 × 5 × 5, 6 × 6 × 6 and 7 × 7 × 7 IS = 1 (a single **k** point, the Γ point) was used.
  So from 5 × 5 × 5 on the compensating effect of the reduction of the number of **k** points is lost.
2 The figure shows that the exchange cost is dominating in the interval 1 × 1 × 1 to 5 × 5 × 5. In Figure 2 the points are fitted with a polynomial of third order, ax + bx^2^ + cx^3^. The fitting (7 points for 3 parameters) is quite accurate, and confirms that the trend for the exchange cost for very large cells scales with the third power.
3 The Coulomb and Ewald (the two components, short and long ranged, of the treatment of the Coulomb series) costs grow very slowly, also in the right side of the curve, confirming that this part of the code is extremely efficient.^64–66^ A pure DFT calculation permits to separate the exchange cost from the Coulomb one; for the 3 × 3 × 3 supercell, the cost is 13.8 s (Coulomb) vs 73.6 s (exchange) to give the total of 87.4 s of BIELET in column 3.
4 Also the PG curve increases rapidly from 6 × 6 × 6 on, nearly as rapidly as FK. This is due to the large number of direct space vectors involved in the Fourier transform (see eq 2), but might also reflect some inefficiency in the parallelization of this part of the code.
5 The effect of symmetry on BIELET can be appreciated comparing column 3 and 9 (4 vs 8 symmetry operators): it goes down from 87 to 35 s. In general, symmetry can reduce dramatically the cost of diagonalization: for example in the case of icoshaedral fullerenes,^84^ thanks to the very high number of point symmetry operators (120, with irreducible representations, IRREP, of dimension up to five; we remind that the factorization according to the various rows of the IRREP permits to diagonalize only one block, and to generate the other blocks by rotation) it has been possible to optimize the structure of a giant fullerene containing 6000 carbon atoms on a single processor in less than 24h.

Overall, Table 6 and Figure 2 show that it is possible to compute supercells with up to 3430 atoms, described by an *all electron* basis set, for an open shell system (with then double diagonalization for each **k** point) with extremely high accuracy (see the ETOT/f.u. row in the table).

## 5 Results: Hartree–Fock

We start the analysis from the pure HF results. In Tables 7, 8 and 9 the effect of the tolerance Tx≡T3, T4, T5/2 on the five quantities mentioned in the Introduction, and referring to KCrF_3_, is documented: the total energy, the equilibrium geometry, the energy difference between three competing structures and between the ferromagnetic (FM) and antiferromagnetic (AFM) solutions, and finally the vibrational spectrum (wavenumbers and IR intensities).

**Table 7:** Effect of the Exchange Tolerances on the Total Energy Obtained with SG 12, 140, and 127 for KCrF_3_ (Hartree–Fock, FM Solution)^a^

Space group	Tx = 6	Tx = 8	Tx = 10	Tx = 12	Tx = 16	Tx = 22	δE (6)	δE (10)	δE (16)
12 FM	–0.268853	–0.271976	–0.272849	–0.274007	–0.274057	–0.274160	+5307	+1311	+103
140 FM	–0.267456	–0.271558	–0.272608	–0.273731	–0.273784	–0.273883	+6427	+1275	+99
127 FM	–0.267440	–0.271541	–0.272593	–0.273717	–0.273771	–0.273868	+6428	+1275	+97
12 (cell × 8)	-	-	–0.272840	-	-	-	-	-	-
ΔE(140–12)	+1387	+418	+241	+276	+273	+277	+1110	–36	–4
ΔE(127–12)	+1413	+435	+256	+290	+286	+293	+1120	–37	–7
ΔE(127–140)	+16	+17	+15	+14	+13	+15	+1	0	–2

a The unit cell in the three cases contains 10 atoms. The δE­(X) columns give the difference (in μE*_h_*) between the total energies obtained with Tx = 6, Tx = 10, Tx = 16, and Tx = 22. The ΔE rows give the energy differences (in μE*_h_*) between the three phases. To the total energies shown in the table, −3882 E*_h_* must be added.

The aim of this large set of properties is to show that the present scheme for treating HFX provides numerically stable results for the most important ground state properties, involving the total energy and the total energy differences, the total energy first derivatives (equilibrium geometry) and the total energy second derivatives (vibrational frequencies).

In the next section this evidence will be extended to hybrid functionals, with any percentage X of HFX, where the smooth behavior of the properties with respect to X will be documented.

### 5.1 The Absolute Value of the Total Energy

As regards the first point, the total energy of KCrF_3_ has been evaluated with Tx = 6, 8 (poor conditions), 10, 12 (good), 16 and 22 (extremely accurate conditions) at the geometry optimized with Tx = 10. Tx = 16 and 22 are inserted in the list for two reasons: (i) to show that nothing dramatic happens from a computational point of view when Tx is pushed to very high values; ii) to show that the convergence with respect to Tx is smooth also when an increasingly high number of exchange bielectronic integrals is included in the calculation. For ease of discussion, the effect of the three Tx tolerances is not discussed separately here. We remind that the number of point symmetry operators in the tetragonal and monoclinic SG is different (16 and 4, respectively).

The δ columns of Table 7 provide the total energy difference (in μE_*h*_) between the indicated Tx value and Tx = 22, taken as a reference, being formally the *converged* result. The total energy as a function of Tx varies quite regularly, and the differences become smaller and smaller: they are of the order of 6 mE_*h*_ (6000 μE_*h*_) for Tx = 6, become about 6 times smaller (then 1 mE_*h*_) for Tx = 10, and then 1 order of magnitude smaller for Tx = 16 (0.1 mE_*h*_). So, the default value we used in this paper (and in many other papers published in recent years) is affected by a convergence error for the exchange series of the order of 1 mE_*h*_, a number that might create problems, in principle, when optimizing the geometries (see below) and/or computing the relative stability of different phases. We show in the following that this is not the case, because:

1 In performing energy differences, the largest part of the convergence error with respect to Tx cancels.
2 The FIXINDEX directive (see the previous section) smooths the largest part of the discontinuities in the potential energy surface.

We recall that in Table 6 it has been shown that the total energy of a supercell containing 3430 atoms can be computed (with high accuracy) on a small parallel machine, using only 64 processors.

### 5.2 The Relative Stability of Three Different Phases

The Δ rows in Table 7 provide the energy differences between the three phases (each one at the geometry optimized with Tx = 10). At Tx = 10, Δ(140–12) becomes +241 μE_*h*_; from Tx = 12, it increases by approximately 35 μE_*h*_, from 241 to about 275 μE_*h*_, with oscillations of the order of 4 μE_*h*_; the behavior for the other two Δ rows is similar. It is worth noting that the difference between SG 127 and SG 140 is in all cases between 13 and 17 μE_*h*_, the reason being that the structures obtained with SG 127 and 140 are very similar, as they correspond to the *twisted* or *untwisted* sequence of *ab* planes along *c* (see Figure 1), and the interaction between planes turns out to be extremely small. The above numbers confirm that the energy difference between different phases is stable with respect to the Tx parameter.

### 5.3 The Optimization of the Geometry

In Table 8 we superpose the effect of the geometry optimization to the effect of Tx. In the first row the total energy of the FM solution as a function of Tx is shown. All the calculations are performed at the geometry optimized with Tx = 10. In the second line, for each Tx value the geometry is reoptimized. It is clear that the two Tx = 10 energies coincide, by construction. They coincide also for Tx = 8. In the other cases the difference is extremely small; for example just 6 μE_*h*_ are gained for Tx = 12 and 14. For the relatively poor Tx = 6, the difference increases to 48 μE_*h*_.

**Table 8:** Effect of the Exchange Tolerances and of the Optimization of the Geometry on the Total Energy of KCrF_3_ (HF, SG 127) for the FM and Various AFM Solutions^a^

	6	8	10	12	14
FM*	–0.267440	–0.271541	–0.272593	–0.273717	–0.273736
FM OPT	–0.267488	–0.271541	–0.272593	–0.273723	–0.273742
δ	–48	0	0	–6	–6
AFMA OPT	–0.267503	–0.271560	–0.272608	–0.273741	–0.273747
ΔE(AFMA – FM)	–15	–19	–15	–18	–5
AFMC OPT	–0.266499	–0.271313	–0.272350	–0.273482	–0.273501
ΔE(AFMC – FM)	+989	+228	+243	+241	+241
AFMG OPT	0.267319	–0.271337	–0.272371	–0.273508	–0.273525
ΔE(AFMG – FM)	+121	+204	+222	+215	+217
ΔE(AFMG – AFMA – AFMC)	–853	–5	–6	–8	–19

a In the FM line, the energies refer to the geometry optimized with Tx = 10 (this is the meaning of the star). In the FM OPT line, for each case the geometry is reoptimized. To the energies shown in the table, −3882 E*_h_* must be added. The total energies refer to two formula units. ΔE gives the energy difference between the FM and the AFM solutions, obtained by doubling the cell along *c*. The cell contains then 4 f.u., 20 atoms, 4 Cr ions. The AFMA, AFMC and AFMG solutions have been obtained by inverting the spin of 2 out of 4 Cr ions in the cell. In all cases the geometry is optimized. Under the hypothesis of additivity and of first neighbors only interactions, ΔE­(AFMA – FM) = 4J_1_, ΔE­(AFMC – FM) = 8J_2_, ΔE­(AFMG – FM) = 4J_1_ + 8J_2_, where J_1_ is the single interaction along *c*, and J_2_ in the *ab* plane.

The equilibrium geometry (not shown) for the various Tx values is essentially the same, as the extremely small energy changes in the Table confirm. In other words, working with Tx = 8, or 10, or 12, or 14 produces essentially the same equilibrium geometry.

### 5.4 The Energy Difference FM-AFM

In order to gain the freedom necessary for separately reversing the spins along *c* (AFMA), or in the *ab* plane (AFMC), or on all the first six Cr neighbors (AFMG), we doubled the cell along *c*. In this new cell, with 4 f.u., 20 atoms, there are 4 Cr ions, whose spin can be reversed in different ways. In Table 8 the results of the optimization of AFMA, AFMC and AFMG are shown.

It is well-known^54–58^ that KCrF_3_ is a one-dimensional antiferromagnet along the *c* direction, whereas in the *ab* plane the ferromagnetic solution is more stable than the AFM one. Our simulation shows that AFMA is more stable than FM by 15, 19, 15, 18, and 5 μE_*h*_ when Tx increases from 6 to 14. For AFMC, the FM solution turns out to be more stable by 228, 243, 241, and 241 μE_*h*_ for Tx from 8, 10, 12 and 14; for the value Tx = 6, it is about four times larger, 989 μE_*h*_, confirming that Tx = 6 is too poor. These numbers are extremely small. It is well-known that HF largely underestimates the spin–spin interactions, the HF d electron charge distribution being very localized, and then the overlap with the p electrons of F becomes very small. In a previous paper^80^ by some of the present authors, and devoted to KMnF_3_, KFeF_3_, KCoF_3_ and KNiF_3_, it is shown that HF provides an FM-AFMG energy difference which is 6 to 7 times smaller than the B3LYP one. When compared to experiment, B3LYP overestimates the FM-AFM energy difference, HF underestimates it by a factor 3 to 4 (see Table 6 of ref 80). A similar underestimation was observed for KCuF_3_:^85^ the magnetic exchange constant *J*, when computed at the HF level, is about 25% (46 vs 200) of the experimental one (see Table 3 of ref 85).

Under the hypothesis of additivity, already verified in previous papers, and limiting the interactions to the first six neighbors, the AFMG stabilization energy is expected to be the sum of the stabilization energy of AFMA and AFMC. The table shows that this condition is satisfied with a residual difference of less than 20 μE_*h*_, excluding again the lowest Tx value.

### 5.5 The Vibrational Spectrum

In Table 9 the vibrational wavenumbers and IR intensities are reported for the structure in SG 140 as a function of Tx, for Tx = 6 (poor accuracy, as already discussed for the previous tables), Tx = 10 (the default value) and Tx = 16 (high accuracy). The lowest energy structure of KCrF_3_ at low T is monoclinic,^54–58^ whereas the tetragonal phase is more stable between 80 and 290 K. The quantum mechanical calculations are performed, obviously, at T = 0 K, so that if the tetragonal symmetry is imposed, the instability produces imaginary wavenumbers (indicated as negative in Table 9). It is well-known that, in the diagonalization process of the dynamical matrix, the wavenumbers more sensitive to the numerical noise are the ones close to zero. Table 9 shows that the negative eigenvalues, and the ones below 110 cm^–1^, show the maximum difference between Tx = 6 and 10, which remains however always smaller than 3 cm^–1^. Above 110 cm^–1^, the maximum difference never exceeds 1.5 cm^–1^. The statistical indices, in the last three lines, indicate that, for Tx = 6, MAX (maximum absolute difference with respect to Tx = 16) = 2.3 cm^–1^, and MEAN (mean absolute difference with respect to Tx = 16) = 1.18 cm^–1^. For Tx = 10, these numbers reduce to 1.83 and 0.45 cm^–1^. The IR intensities follow the trends of the wavenumbers.

**Table 9:** B3LYP Wavenumbers (in cm^-1^) and IR Intensities (in km/mol) of the FM Tetragonal Phase of KCrF_3_ (Space Group $I\frac{4}{m}\mathrm{mc}$ , N. 140) as a Function of the Tolerance Tx for the Exchange Series, with Tx = T3 = T4 = T5/2^a^

		Tx = 6		Tx = 10		Tx = 16		Tx = 10; no symmetry	
N	IRREP	ν	I(IR)	ν	I(IR)	ν	I(IR)	ν	I(IR)
1–2	E_*g*_	–41.83	I	–42.90	I	–41.25	I	–42.68; −42.25	I
3	B_1*g*_	–38.29	I	–41.20	I	–39.44	I	–42.25	I
4–5	E_*u*_	0.00	0.0	0.00	0.0	0.00	0.	0.0 (2)	0. (2)
6	A_2*u*_	0.00	0.0	0.00	0.0	0.00	0.	0.0	0.
7–8	E_*u*_	100.44	255.79	97.96	256.8	98.14	257.16	97.93 (2)	128.4 (2)
9–10	E_*g*_	103.74	0.0	101.27	I	101.45	I	101.27 (2)	I
11	A_2*u*_	104.33	110.97	102.08	111.8	102.41	111.85	102.08	111.7
12	A_2*g*_	110.06	0.0	107.81	I	107.88	I	107.76	I
13–14	E_*u*_	155.82	1.6	155.72	1.5	154.56	1.56	155.62; 155.64	0.75 (2)
15–15	B_1*u*_	158.51	0.0	157.34	I	157.22	I	157.35	I
16	A_2*g*_	242.44	0.0	243.00	I	241.17	I	242.72	3.47
17–18	E_*u*_	245.22	202.57	244.36	198.0	244.42	199.79	244.21; 244.29	98.0; 94.7
19	A_2*u*_	253.21	156.23	252.66	154.8	252.09	154.18	252.66	154.7
20–21	E_*u*_	266.41	154.11	266.33	159.0	265.56	154.53	266.07; 266.19	74.1; 80.0
22	B_2*g*_	267,30	0.0	266.94	I	267.27	I	267.77	6.55
23–24	E_*g*_	269.31	0.0	268.69	I	268.00	I	268.68 (2)	I
25	B_1*u*_	320.67	0.0	320.39	I	320.37	I	320.39	I
26	A_1*g*_	368.85	0.0	367.41	I	368.10	I	369.08	I
27–28	E_*u*_	475.02	493.21	473.73	494.1	473.53	496.62	474.27; 474.30	246.9; 247.0
29–29	A_2*u*_	511.22	293.02	511.07	293.91	511.00	294.69	511.06	293.61
30–30	B_2*g*_	535.82	0.0	535.51	I	535.11	I	535.75	I
	MEAN	1.18		0.45		–		0.24	
	MAX	2.30		1.83		–		1.67	
	RMS	0.49		0.38		–		0.23	

a In the last two columns the symmetry has been removed. Then the degenerate modes of E*_g_* or E*_u_* symmetry might split to some amount. When this is the case, two wavenumbers and intensities are reported. I stands for inactive. In the three bottom lines, three statistical indices are applied to the wavenumbers. MEAN is the mean value of the absolute difference between the Tx = 6 or Tx = 10, and the Tx = 16 columns, the latter containing the most accurate result. MAX is the maximum absolute difference, and RMS is the root mean square, again applied to the absolute differences. The statistical indices in the last columns refer to the comparison of the Tx = 10 wavenumbers with and without symmetry.

The last column provides an additional check of the accuracy of the frequency calculation. We must underline that in the construction of the Hessian matrix, the point symmetry is fully exploited, as discussed in ref 74 (see also ref 86); as a consequence, the SCF+G (gradient, see the Computational Section) calculations, that should be 3 × 10 + 1 = 31 (3 coordinates times 10 atoms plus the equilibrium position) when symmetry is not exploited, reduce to 9 SCF+G calculations with a different number of point symmetry operators: 16 in the central point, 2 for 5 displacements, and 4 for 3 displacements.

Obviously the number of computed integrals (in the case under discussion here, the exchange bielectronic integrals) is quite different if the number of point symmetry operators is 16 or 2. This imposes high accuracy in the selection of the integrals and in their evaluation.

We can now look at the last two columns of Table 9, obtained by removing all symmetry operators (apart from identity), and using Tx = 10. When symmetry is not imposed, the degeneracy can be lost: note however that the maximum splitting is much smaller than 1 cm^–1^, and that the difference between the second and fourth columns is extremely limited. The IR intensities are slightly more sensitive, with differences in the low intensities which can reach 3–4%. The statistical indices confirm that the calculation with and without symmetry produce essentially the same numbers (wavenumbers and intensities).

For space reasons, the Raman intensities are not reported. However, the same comments we did above for the IR intensities, apply also to the Raman intensities.

## 6 Results: From Pure Hartree–Fock to Hybrids

In this section we move from pure Hartree–Fock to hybrid functionals, such as PBE0,^3^ B3LYP,^1,2^ HSE06,^5^ characterized by a variable percentage of HFX in the exchange part of the XC (exchange-correlation) term. In order to explore with continuity the effect of the HFX percentage, we employ a fairly new functional, PBE­(X),^53^ such that PBE(0) = PBE and PBE(25) = PBE0. The dependence of a set of properties (band gap, density matrix, Jahn–Teller splitting, orbital ordering, equilibrium geometry, FM-AFM energy difference) on X, or, more generally, on the adopted functional, is analyzed.

We start from the band gap. In Figure 3, both the majority (α) and the minority (β) gaps are shown for KCrF_3_ (SG 140, FM solution). Starting from X = 100, the two gaps decrease linearly (or nearly linearly) from 13.99 (α) and 18.85 eV, to zero at X = 0. The real discontinuity in the two curves is between X = 3 and 4, when the system becomes metallic. Also the B3LYP, HSE06 and HF gaps are shown in the figure. We remind that the ingredients of the B3LYP functional (characterized by X = 20), differ in many respects from the PBE0 ones. In spite of that, the B3LYP gap is very close to the PBE(20) one (2.67 vs 2.75 eV for the α curve, only 0.08 eV (3%) difference). The differences between the HF and PBE0 Hamiltonians are even larger than the ones between B3LYP and PBE0. However, the PBE(100) gap differs from the HF one by only a fraction of one eV (13.70 vs 13.84, and 19.57 vs 18.90 eV for the α and β curve, i.e., about 1.0 and 3.5%). In the figure, the HSE06 gap has been located at X = 25. This is somehow arbitrary, HSE06 being a *range separated*, not a *global* hybrid, so that only at *short-range* the HFX, with X = 25, is adopted.

**Figure 3:**
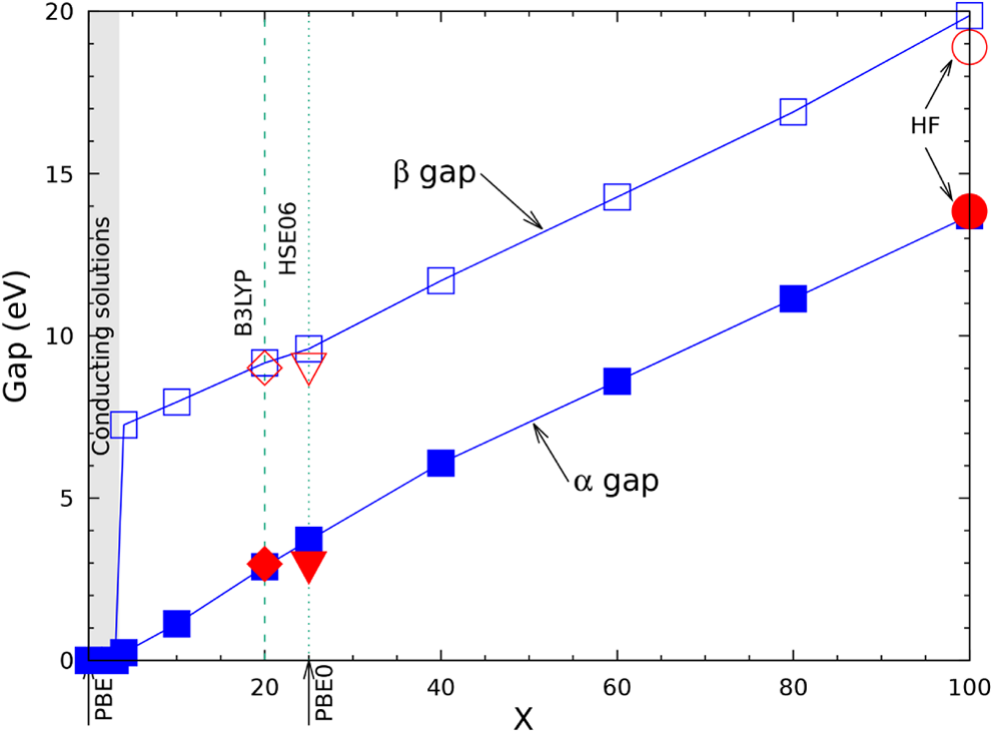
The band gap of KCrF_3_ perovskite (ferromagnetic solution for the tetragonal space group I4/mcm) as a function of X, the Hartree–Fock exchange percentage in the PBE­(X) functional. When X = 0, PBE­(X) coincides with PBE; when X = 25, it coincides with PBE0. The two curves refer to the majority (filled symbols) and minority (unfilled symbols) spin, respectively. The red symbols indicate the HF (at X = 100), HSE06 (X = 25) and B3LYP (X = 20) results.

On the basis of Figure 3 and of the above comments, we can guess that any functional with X larger than, say, 5, produces a non metallic solution for KCrF_3_, FM. More important, the comparison of the PBE(100) gap with the HF one, of the PBE(20) with the B3LYP one, and also of the PBE(25) with the HSE06 one, indicates that the gap in this system (but the generalization to any insulator is obvious) is totally determined by the HF exchange. All other ingredients of the functionals have an extremely limited influence on the gap.

We can ask whether other properties depend dramatically on X as the band gap does. We examine first the Jahn–Teller splitting and deformation. In Figure 4 the total energy differences between a here additionally introduced cubic structure (SG 221), the tetragonal structure (SG 140), and the monoclinic structure (SG 12) are shown. We remind that at low temperature the KCrF_3_ structure is monoclinic; it becomes tetragonal above 150 K. In the cubic symmetry, the two e_*g*_ orbitals are symmetry related, and are then forced to remain degenerate. When reducing the SG to 140, the *c* lattice parameter is no more symmetry related to the *a* and *b* lattice parameters, and $d_{z^{2}}$ is no more symmetry related to $d_{x^{2}-y^{2}}$ . In SG 12 the octahedra remain essentially the same as for SG 140, but slightly rotated.

**Figure 4:**
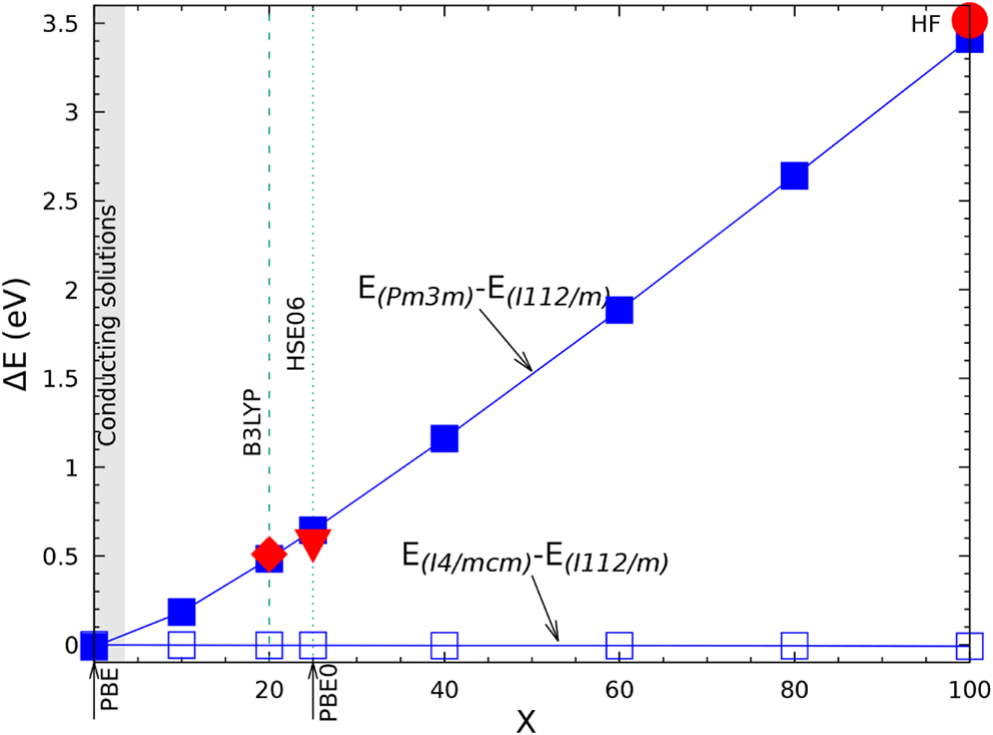
Total energy difference (top curve: SG *Pm*-3m minus SG I112/m; bottom curve: SG I4/mcm minus SG I112/m) for KCrF_3_, as a function of the Hartree–Fock exchange percentage X in the functional PBE­(X). In the cubic case, the solution is metallic, the two e*_g_* orbitals are symmetry related, and their occupancy is 0.5 |*e*| each. In SG I4/mcm (2 f.u. in the unit cell, as in SG I112/m), both the Jahn–Teller splitting and the orbital ordering are allowed. In SG I112/m also the octahedra rotation is possible. The energy difference between SG I4/mcm and SG I112/m is very small in this scale, and is expanded in the next figure. The red symbols indicate the HF (at X = 100), HSE06 (X = 25) and B3LYP (X = 20) results.

The two curves in Figure 4 are very regular, confirming that the optimization process is very accurate.

As for the gap, the Jahn–Teller effect on the total energies (see the two upper curves) decreases nearly linearly from X = 100, where it is 3.5 eV large, down to zero for X < 4. The red symbols refer to the HF, HSE06 and B3LYP values, which are very close to PBE(100), PBE(25) and PBE(20). The figure shows that the Jahn–Teller effect on energy is much larger than the difference between SG 140 and SG 12; the latter being the energy gain due to the rotation of the octahedra, allowed in SG 12, but not in SG 140. In the scale of the figure, this difference appears as close to zero; for this reason the same energy difference between the two SGs is shown in Figure 5 on a different scale, and compared with other functionals, characterized by a different amount of HFX: HF (100%), WC1LYP^87^ (33.33%), PBE0 (25%), HSE06 (25% at short-range), B1WC^88^ (16%). In all cases the energy of SG 12 is lower than the one of SG 140. The results differ however from each other on this tiny scale (just 8 meV). The HF, HSE06 and PBE0 results are very similar (3.29, 3.55, 3.55 meV respectively); B3LYP increases to 5.57 meV, about the same value of B1WC; WC1LYP produces the lowest difference, just 0.40 meV. For this property, the role of X is not so dominant as in the previous figure, and the other component of the functionals produce large differences: for example PBE(100) differs from HF by 5.10 meV, and PBE(20) from B3LYP by 2.17 meV. Limiting the comparison to the three functionals that the present authors consider the most reliable in the above list, PBE0, HSE06, B3LYP, the relative stability of SG 12 with respect to SG 140 is in between 3.55 and 5.57 meV.

**Figure 5:**
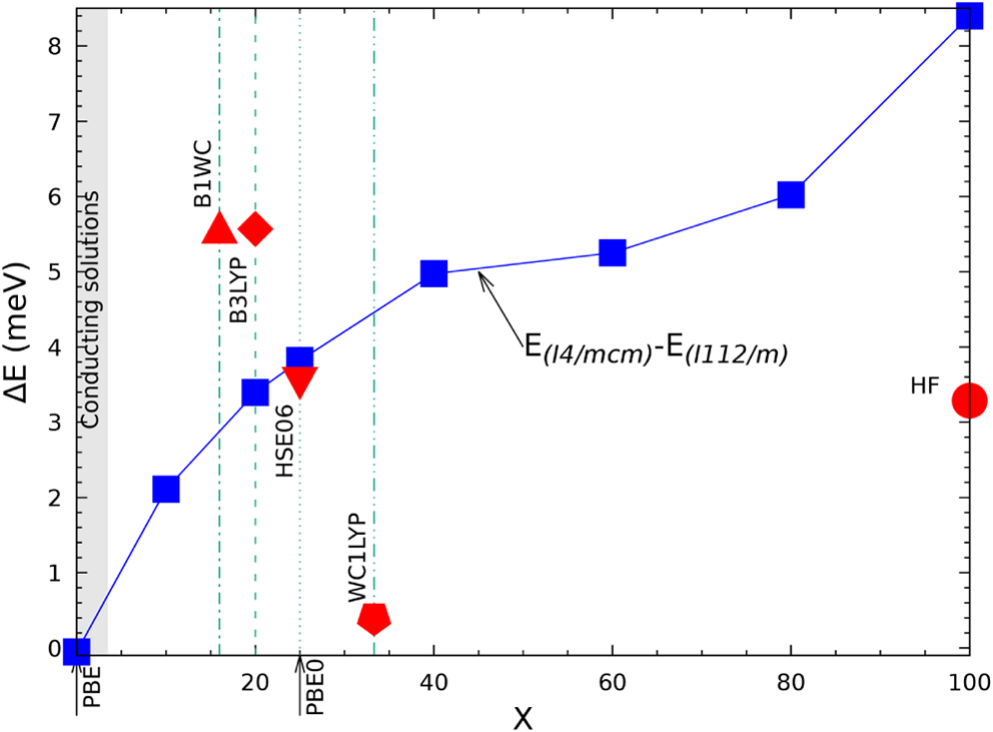
The total energy difference between the tetragonal and the monoclinic solutions (SG I4/mcm and I112/m) for KCrF_3_, FM, for various functionals. The continuous curve is the PBE­(X) difference, as a function of X. The red symbols refer to HF­(X = 100), WC1LYP­(X = 33.33), HSE06­(X = 25, square), PBE0­(X = 25), B3LYP­(X = 20) and B1WC­(X = 16). In the tetragonal case, the Jahn–Teller deformation and the orbital ordering are allowed. In the monoclinic case, the octahedra are also allowed to rotate.

In Figure 6 the three Cr–F distances in the octahedron are shown. The data for PBE0, HSE06 and B3LYP are very similar: 1.99, 1.99, and 2.00 Å for Cr–F_*a*_, and 2.32, 2.32, and 2.34 Å for Cr–F_*b*_, the two distances in the *ab* plane. The Cr–F distance along *c* is 2.03, 2.04, and 2.05 Å in the three cases. The anisotropy in HF is slightly smaller, with the three distances at 2.04, 2.30, 2.07 Å. The HSE06 and B3LYP distances are very close to the PBE(25) and PBE(20) ones, whereas the PBE(100) values are far from the HF ones, overestimating the reduction of the deformation of the octahedron, which is a consequence of the Jahn–Teller distortion. At the metallic limit (X < 4), there is no Jahn–Teller splitting and orbital ordering, *a* and *c* are the same, the three Cr–F distances are the same, and the cell is cubic.

**Figure 6:**
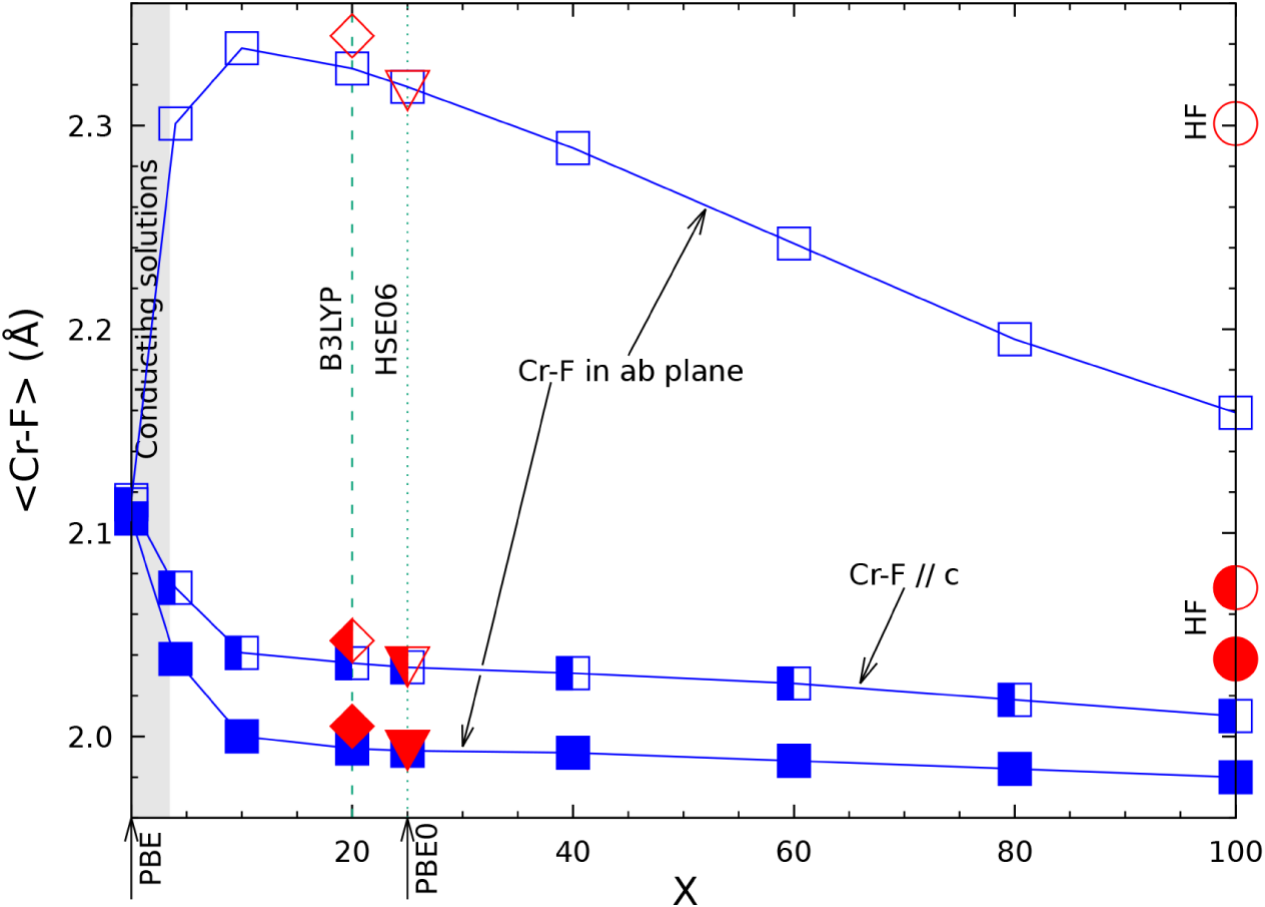
The variation of the three Cr–F distances as a function of X, the HF exchange percentage in the PBE­(X) functional. The top and bottom curves (empty and full squares), refer to the distances in the *ab* plane. The central curve, to the distance along the *c* direction. The red symbols correspond to the HF, HSE06 and B3LYP results. The system becomes metallic at X = 3.5, where the differences between the three Cr–F distances disappear.

As a last example, we show in Figure 7 the energy difference between the FM solution and the AFMA and AFMC solutions for SG I4/mcm as a function of X in PBE­(X) and for B3LYP, HSE06, PBE0 (=PBE(25)) and HF (red symbols). We remind that in AFMC the spin of the four first Cr neighbors of the reference Cr ion in the *ab* plane is reversed, whereas in AFMA the spin of the first two Cr neighbors along *c* is reversed. The FM energy is always lower than the AFMC one, and higher than the AFMA one, when X is larger than, say, 10, confirming that there is ferromagnetic order in the *ab* plane. Below this value, the solution becomes metallic. Both curves increase in magnitude when X goes from 100 down to lower values, although the slope is larger in the negative (AFMC) branch. Note that the FM-AFMA differences are much smaller than the FM-AFMC, and are multiplied by 10 in the figure, to keep the same scale for the two curves. The HSE06, B3LYP and PBE0 values are very similar (2.5, 3.0, 2.89 meV for FM-AFMA, and −15.4, −13.3, −14.4 meV for FM-AFMC); B3LYP is relatively close to PBE(20) (3.04 and 2.91 for FM-AFMA, and −13.3 and −18.3); HF, on the contrary, is quite far from PBE(100) in both the positive and negative curve (0.22 vs 1.3 meV; −2.93 vs −1.41 meV).

**Figure 7:**
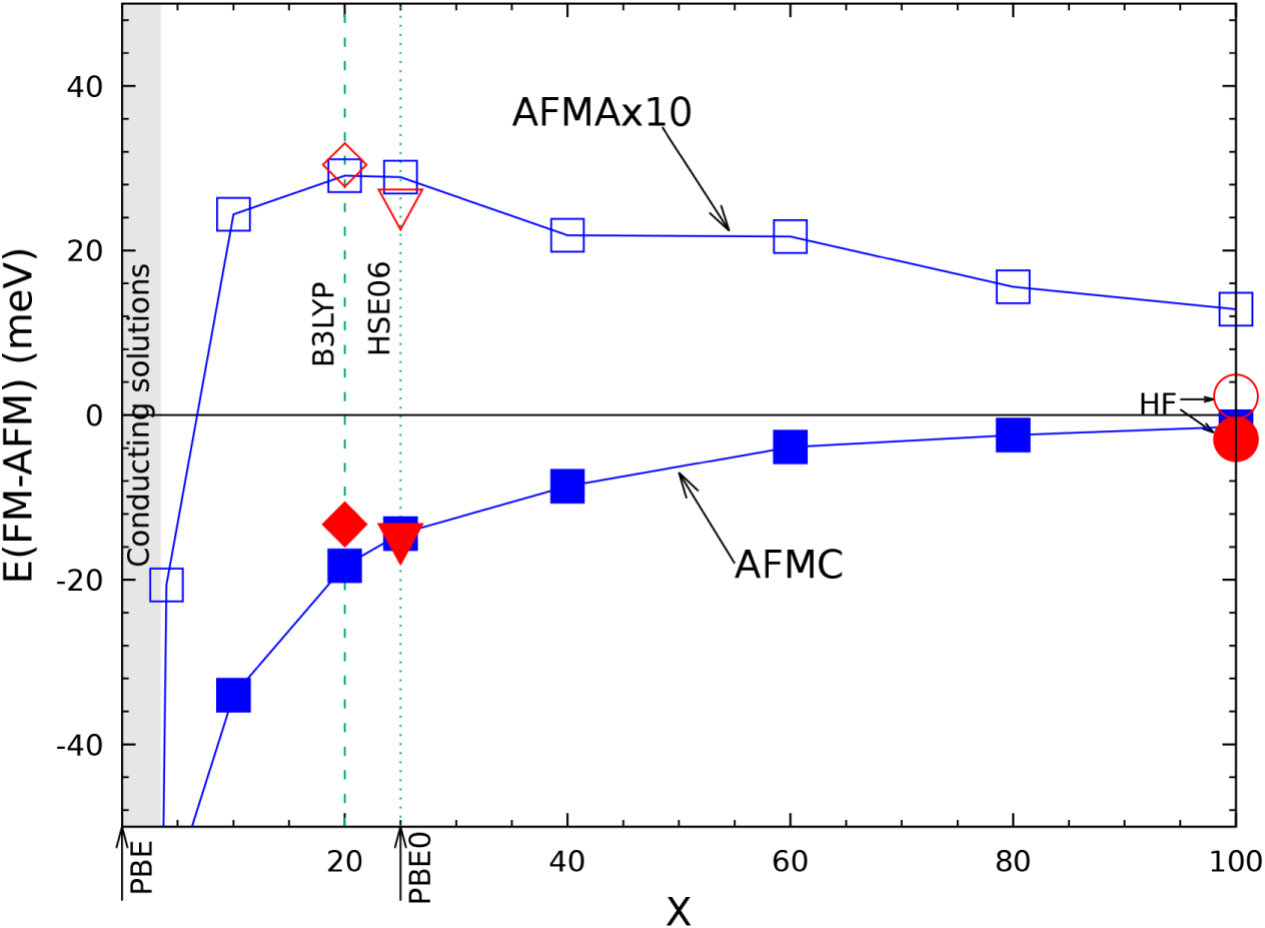
The energy difference (SG I4/mcm) FM-AFMA (top curve; the spin is inverted on the two Cr ions first neighbors of the central Cr ion along *c*) multiplied by 10 to improve its visibility and FM-AFMC (bottom curve; the spin is inverted on the four Cr first neighbors in the *ab* plane of the central Cr ion) as a function of the HF exchange percentage X. The FM energy is lower than the AFMC one, and higher than the AFMA one. The HF, HSE06, and B3LYP results are also shown as red circles, squares, triangles. PBE0 coincides with PBE (25). It should be noticed that the energy scale is about 6 times larger than in Figure 5, and 70 times smaller than in Figure 3.

Overall we observe that the HF exchange percentage does not have the same dominant role as it had for the band gap in Figure 3, and for the effect of the Jahn–Teller distortion in Figure 4, similarly to what happens for the stability between SG 140 and SG 12 (see Figure 5). It should be noticed, however, that the energy scale in Figure 5 is about six times smaller than in Figure 7, and about 500 times smaller than in Figure 3. So, on a very expanded scale, as in Figure 5, the other components of the functionals (for example the correlation term) become important. As regards the absolute values of the energy differences between AFMC or AFMA and FM, they are very small for HF or PBE(100), because at this extreme the d shell on Cr is contracted and closer to the nucleus; when X decreases, the Cr–Cr interaction through the F ions increases, and at PBE(25) it is 1 order of magnitude larger than for HF, in line with previous results.^80^

## 7 Conclusions

We can summarize the main results of the present study as follows:

1 It is possible to perform Hartree–Fock calculations for 3D periodic systems, with high accuracy and low cost, with the CRYSTAL code.
2 In the present study we document more explicitly the scheme for the calculation of the HF exchange series, with reference to KCrF_3_. The scheme is very effective: unit cells with 4 f.u. can be optimized on a single core laptop, and supercells containing 3430 atoms can be run on 64 processors of a parallel machine. We also document the smooth behavior of many properties (band gap, equilibrium geometry, Jahn–Teller splitting, FM-AFM energy difference) when the percentage of HFX has been varied from 100 to 0. It is shown that some of the properties are very sensitive to HFX, for example the band gap and the FM-AFM energy difference. The present results, we hope, clearly document the crucial role of HFX, with respect to other components of the functional (correlation energy), frequently indicated as very important for determining the magnetic properties.
3 A very large fraction of simulations of this kind of systems (first row TM perovskites) is performed with LDA+U and GGA+U. Apparently, this choice is related to the difficulties of implementing functionals containing a fraction of HFX. This is certainly true when a plane wave basis set is used,^52,89,90^ but also with a Gaussian basis^51^ the numerical problems are apparently not always solved. The analysis of the data in the literature seems to suggest, however, that LDA+U or GGA+U are able to open the gap with respect to pure LDA and GGA, but we have not been able to find any evidence that the subtle effects of the exchange term on many properties can be simulated by U, which is in most of the cases used as a parameter for reproducing some of the experimental results.

In summary, the Hartree–Fock exchange treatment within the CRYSTAL code for a delicate system such as KCrF_3_ is not only possible at low cost, but is also very stable with respect to the computational parameters.
